# Revealing the Pharmacological Mechanism of Tibetan Medicine Wugeng San in Treating Rheumatoid Arthritis Through an Integrated Strategy of Chemical Composition Analysis, Network Pharmacology, Machine Learning, and In Vivo Experiments

**DOI:** 10.3390/ph19050718

**Published:** 2026-04-30

**Authors:** Zixian Chen, Yu Zhang, Shuangqi Chen, Chunxia Zhang, Rui Gu, Shaohui Wang

**Affiliations:** 1 State Key Laboratory of Southwestern Chinese Medicine Resources, School of Ethnic Medicine, Chengdu University of Traditional Chinese Medicine, Chengdu 611137, China; chenzixian@cdutcm.edu.cn (Z.C.); 3208546220@qq.com (S.C.); 2 State Key Laboratory of Southwestern Chinese Medicine Resources, School of Pharmacy, Chengdu University of Traditional Chinese Medicine, Chengdu 611137, China; 3326851741@qq.com; 3 Qingdao Academy of Chinese Medicinal Sciences, Shandong University of Traditional Chinese Medicine, Qingdao 266114, China; 18202669277@163.com

**Keywords:** Wugeng San, rheumatoid arthritis, network pharmacology, machine learning, SYK

## Abstract

**Background:** Wugeng San (WGS) is a traditional Tibetan medicinal preparation that has long been used to treat inflammatory and arthritic conditions. However, its contemporary pharmacological validation and the mechanisms underlying its action in rheumatoid arthritis (RA) have not been fully investigated. **Objective:** For the first time, this study aimed to systematically investigate the therapeutic effects of WGS on RA, identify its potential targets, and elucidate its action mechanisms. **Methods:** This study, as the first comprehensive investigation of WGS in RA, employed integrated multiple approaches including chemical component identification via UPLC-Q-TOF/MS, network pharmacology, bioinformatics, machine learning, and in vivo efficacy assessment and mechanism verification in a collagen-induced arthritis (CIA) rat model, a widely accepted experimental model that mimics the key pathological features of RA. **Results:** The results demonstrated that WGS reduced the severity of arthritis in a dose-dependent manner, as evidenced by decreased paw swelling, normalized body weight, and restored levels of pro- and anti-inflammatory cytokines. The high dose of WGS (252 mg/kg) showed an effect comparable to that of methotrexate (0.2 mg/kg). Histological analysis revealed that WGS reduced synovial hyperplasia, cartilage erosion and bone destruction, decreased osteoclast numbers, and promoted osteoblast activity. Eighty-four compounds were identified using UPLC-Q-TOF/MS. Network pharmacology and machine learning analyses indicated SYK as a key target enriched in the NF-κB signaling and osteoclast differentiation pathways. Experimental validation confirmed that WGS suppressed the phosphorylation of SYK and NF-κB pathway components (p65, IκBα, and IKKα/β), decreased MMP1/MMP3 levels, and modulated the Bax/Bcl-2 ratio to promote apoptosis. **Conclusions:** In conclusion, WGS exhibits strong anti-arthritic effects through “multi-component, multi-target, and multi-pathway” mechanisms, likely attributable to the inhibition of the SYK/NF-κB signaling axis, suppression of matrix degradation, and regulation of cellular apoptosis. This research offers a pharmacological basis for repurposing WGS as a promising natural candidate for RA therapy.

## 1. Introduction

Rheumatoid arthritis (RA) is a major challenging disease characterized by symmetrical, polyarticular chronic inflammation [1,2]. The fundamental pathological change is synovial inflammation of the joints. During acute flare-ups, joints swell, exude fluid, and exhibit inflammatory cell infiltration; in the chronic phase, synovial hyperplasia forms vascular membranes, leading to joint bone destruction, deformity, and even permanent disability [3]. The global prevalence of RA, according to epidemiological data [4], is about 1%; the prevalence in China is 0.42%. Epidemiological data indicate that while females have a higher overall prevalence rate, males show a higher annual growth rate of new cases. As a lifelong condition, RA significantly impacts patients’ quality of life, exacerbates the disease burden, and currently has no curative treatment. In spite of the significant side effects of current anti-rheumatics, anti-inflammatories, and immunomodulators, they still relieve the symptoms [5]. Therefore, exploring the potential of traditional medicine in treating RA has great significance.

RA falls under the category of “Zhenbu disease” in Tibetan medicine [6]. According to Tibetan medical theory, health is maintained through the dynamic balance of the “three causes” (Long, Chiba, and Peigen). Under normal physiological conditions, digested food essence is transformed into “yellow water” (a physiological fluid), which regulates body fluids and lubricates joints. However, an improper diet or diminished “stomach fire” (digestive capacity) can lead to incomplete digestion, resulting in the pathological production of turbid yellow water and its excessive accumulation in the joints [7]. Both the “Four Medical Tantras” and the “Tibetan Medical Clinical Notes” emphasize that “wet bi is inseparable from yellow water”, indicating that “Zhenbu disease” is triggered by lifestyle and dietary factors that disrupt the “three causes”. Under the influence of “Long” (one of the three causes), excessive yellow water accumulates in the joints, causing swelling, pain, stiffness, and restricted movement [8].

The Tibetan medicine Wugen San (WGS), also known as the “Five Roots” medicine, is a foundational formula first recorded in the “Diebu Jiyao” and later included in the “Four Medical Classics.” It consists of five herbal components [9]: the dried rhizomes of *Polygonatum cirrhifolium* (Wall.) Royle, the dried tuberous roots of *Asparagus cochinensis* (Lour.) Merr., the dried roots of *Pleurospermum hookeri* C. B. Clarke var. thomsonii C. B. Clarke, the dried roots of *Mirabilis himalaica* (Edgew.) Heim., and the dried mature fruits of *Tribulus terrestris* L. The formula has a sweet taste, mild and balanced nature, and gentle efficacy. It is traditionally used for treating “Long” diseases, regulating the “three causes”, tonifying the body, and drying yellow water (i.e., removing pathological fluid accumulation). WGS is listed in the “Pharmaceutical Standards of the Ministry of Health of the People’s Republic of China Tibetan Medicine Volume” (1995 edition), with its primary function being to dry yellow water for the treatment of cold-type yellow water diseases and joint swelling. Tibetan medical clinical practice has confirmed its efficacy in improving “Zhenbu disease” [10]. However, its current application remains largely experience-based, with a lack of systematic modern pharmacological evaluation and mechanistic evidence, limiting its broader clinical adoption and international recognition. Therefore, the specific role and potential mechanisms of WGS in RA have not yet been systematically explored.

In recent years, artificial intelligence technology has demonstrated broad application prospects in fields such as genomics, proteomics, bioimaging, spatialomics, and drug development, leveraging the advantages of powerful computing power, big data, and language models [11,12,13]. Currently, “AI^+^” technology has become a hot topic in life science research, providing important strategies and research tools for studying the action mechanisms of traditional Chinese medicine (TCM) formulas. It is noteworthy that the application of UPLC-Q-TOF/MS technology enables high-resolution characterization of complex chemical components in TCM, which provides a key method for linking phytochemistry with mechanistic pathway research by network pharmacology and bioinformatics, etc. [14,15]. Therefore, in this study, a comprehensive, innovative approach integrating in vivo experiments, chemical analysis, network pharmacology, bioinformatics, and machine learning was employed to systematically investigate the anti-RA effects of WGS, clarify its potential targets and action mechanisms, provide comprehensive evidence for its anti-RA potential, and promote its clinical application. Furthermore, this integrated approach addresses the current gap in in-depth mechanism research and establishes a template for evaluating traditional formulas using advanced technological platforms.

## 2. Results

### 2.1. WGS Alleviates Arthritis Progression and Modulates Cytokine Expression in CIA Rats

**Figure 1:**
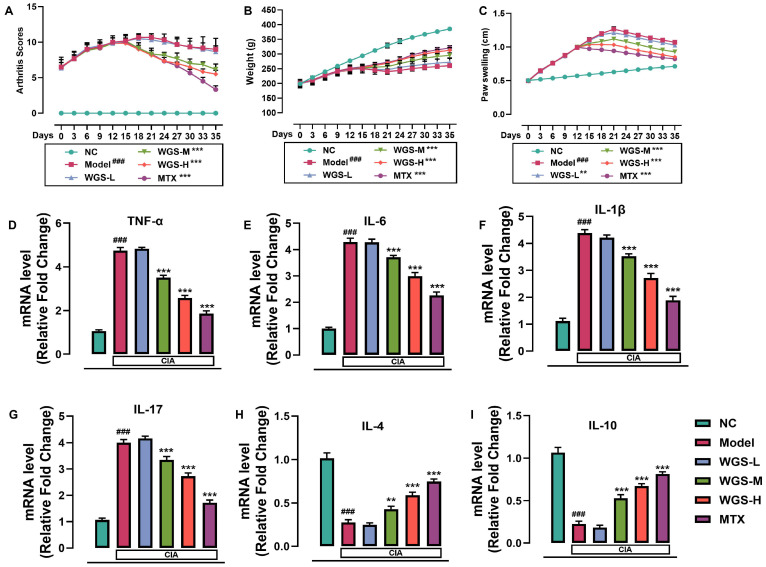
Effect of Wugen San (WGS) on arthritis progression and cytokine expression in a collagen-induced arthritis (CIA) rat model. (**A**) Arthritis scores throughout the experimental period. (**B**) Body weight changes in rats across different groups. (**C**) Paw swelling measurements. (**D**–**I**) Relative mRNA expression levels of cytokines in blood samples: (**D**) TNF-α, (**E**) IL-6, (**F**) IL-1β, (**G**) IL-17, (**H**) IL-4, and (**I**) IL-10. NC: normal control; Model: CIA model control; WGS-L: low-dose Wugen San; WGS-M: medium-dose Wugen San; WGS-H: high-dose Wugen San; MTX: methotrexate (positive control). ^###^ *p* < 0.001 vs. NC; *** *p* < 0.001, ** *p* < 0.01 vs. Model.

To evaluate the therapeutic efficacy of WGS in RA, we first assessed its effects on arthritis progression in the CIA rat model. The results showed that WGS treatment exhibited a dose-dependent attenuation of arthritis progression in the CIA rat model, with comparable effects to MTX. As shown in Figure 1A, arthritis scores in the WGS-treated groups (WGS-L, WGS-M, WGS-H) and the MTX group were significantly lower than those in the model group from day 18 onwards, with the WGS-H group showing the most pronounced effect, with efficacy approaching that of MTX. Body weight changes (Figure 1B) revealed that rats in the model group had stunted growth, while WGS treatment, especially at medium and high doses and MTX administration, partially restored normal weight gain, with WGS-H and MTX groups showing similar weight recovery trends. Paw swelling (Figure 1C) was also reduced in both WGS-treated and MTX groups, indicating alleviated joint inflammation, with WGS-H exhibiting a reduction comparable to that of MTX.

Analysis of cytokine mRNA expression (Figure 1D–I) demonstrated that pro-inflammatory cytokines, including TNF-α, IL-17, IL-6 and IL-1β were upregulated in the model group compared to NC. WGS treatment downregulated these pro-inflammatory cytokines in a dose-dependent manner, with WGS-H and MTX groups showing significant reductions. Conversely, anti-inflammatory cytokines, including IL-4 and IL-10, were downregulated in the model group but were upregulated by WGS treatment and MTX, with the WGS-H group showing increases comparable to those in the MTX group.

### 2.2. WGS Improves Joint Histology and Modulates Cellular Activity in CIA Rats

**Figure 2:**
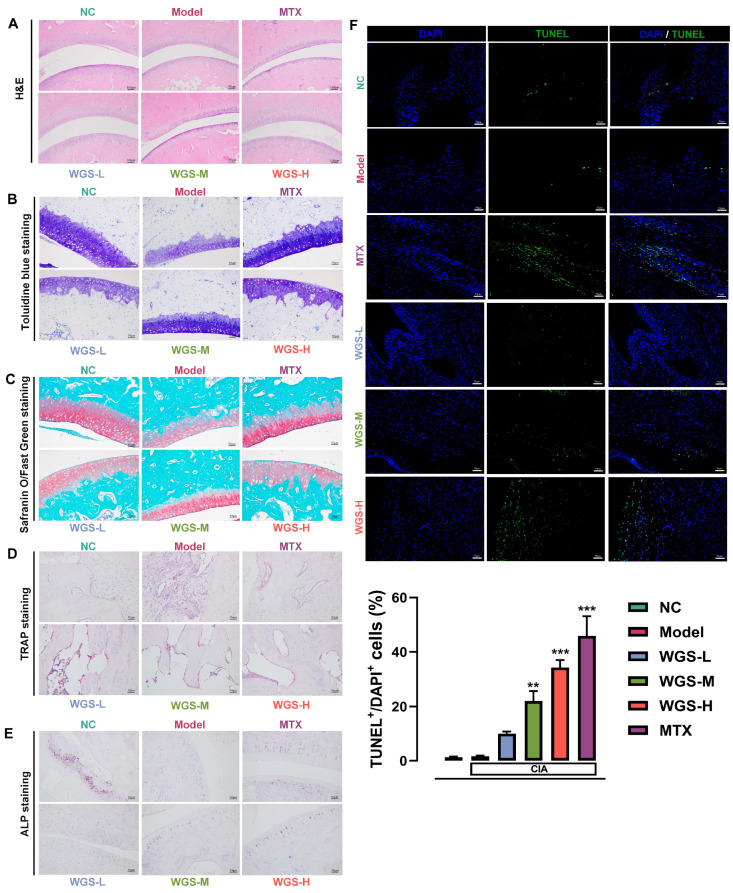
Histological and cellular analyses of joint tissues in different experimental groups. (**A**) Hematoxylin–Eosin (H&E) staining of joint sections (scale bar = 100 μM). (**B**) Toluidine blue staining to assess cartilage matrix (scale bar = 50 μM). (**C**) Safranin O-Fast Green staining for visualizing proteoglycan content in cartilage (scale bar = 50 μM). (**D**) Tartrate-resistant acid phosphatase (TRAP) staining to identify osteoclasts (scale bar = 50 μM). (**E**) Alkaline phosphatase (ALP) staining to detect osteoblast activity (scale bar = 50 μM). (**F**) TUNEL (Terminal Deoxynucleotidyl Transferase Mediated Nick End Labeling) assay for apoptosis detection (green: TUNEL^+^ apoptotic cells; blue: DAPI-stained nuclei) and quantification of TUNEL^+^/DAPI^+^ cells (scale bar = 50 μM). NC: normal control; Model: CIA model control; WGS-L: low-dose Wugen San; WGS-M: medium-dose Wugen San; WGS-H: high-dose Wugen San; MTX: methotrexate (positive control). *** *p* < 0.001, ** *p* < 0.01 vs. Model.

In order to better understand the structural and cellular mechanisms responsible for the therapeutic benefits of WGS in arthritis development and cytokine control, we conducted histological and cellular examinations of joint tissues from CIA rats. Histological analyses (Figure 2) revealed severe joint damage in the model group, including cartilage erosion and bone destruction, as observed by H&E (Figure 2A), Toluidine blue (Figure 2B), and Safranin O-Fast Green (Figure 2C) staining. WGS treatment, particularly at medium and high doses, and MTX treatment preserved cartilage integrity and proteoglycan content, with the WGS-H group showing a protective effect comparable to that of the MTX group.

TRAP staining (Figure 2D) showed a high number of osteoclasts in the model group, which was significantly reduced by WGS treatment and MTX, with the WGS-H group demonstrating a reduction in osteoclast numbers similar to that of the MTX group. ALP staining (Figure 2E) indicated that WGS and MTX enhanced osteoblast activity, as evidenced by increased ALP-positive cells in WGS-treated and MTX groups. TUNEL assay (Figure 2F) revealed that WGS treatment and MTX increased the number of apoptotic cells in joint tissues, with the WGS-H group and the MTX group showing a marked increase in TUNEL^+^/DAPI^+^ cells compared with the model group, suggesting that both treatments may promote the apoptosis of abnormal cells involved in joint inflammation and destruction.

### 2.3. Chemical Composition of WGS

**Figure 3:**
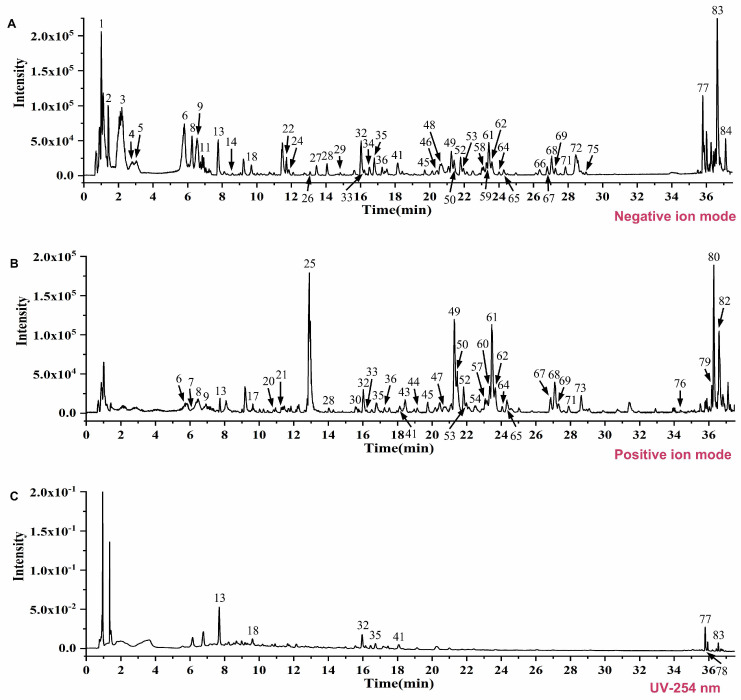
Identification and characterization of chemical components in Wugen San (WGS). (**A**,**B**) Total ion chromatograms of WGS acquired by UPLC-Q-TOF-MS in positive ion mode (**A**) and negative ion mode (**B**). (**C**) UV chromatogram of WGS at 254 nm.

To elucidate the potential pharmacoactive substances responsible for the therapeutic effects of WGS on RA, we performed a systematic identification of the components in WGS, which would facilitate a better understanding of the relationship between the specific active ingredients in WGS and the observed pharmacological effects. The chemical components of WGS were analyzed by using the UPLC-Q-TOF/MS method. Through this analysis, a total of 84 compounds were identified and characterized from WGS (Table S2). The identification process was conducted through a multi-step strategy to ensure accuracy and reproducibility. Initially, mass spectrometry data were compared against the Natural Products HR-MS/MS Spectral Library 1.0 (Shanghai Standard Technology Co., Ltd., Shanghai, China), which contains experimentally acquired reference spectra of authenticated standards. Compounds matching with high confidence, based on mass accuracy, isotopic pattern, MS/MS fragmentation, and retention time, were assigned. For those not present in the library, identification was supported by literature-reported fragmentation patterns and mass spectral interpretation rules. The total ion chromatograms of WGS in positive (Figure 3A) and negative (Figure 3B) ion modes exhibited distinct peaks, indicating the presence of various bioactive compounds. The UV chromatogram at 254 nm (Figure 3C) further characterized the chemical profile, providing a foundation for subsequent network pharmacology studies.

### 2.4. Network Pharmacology Identifies Key Targets and Pathways of WGS in Treating RA

**Figure 4:**
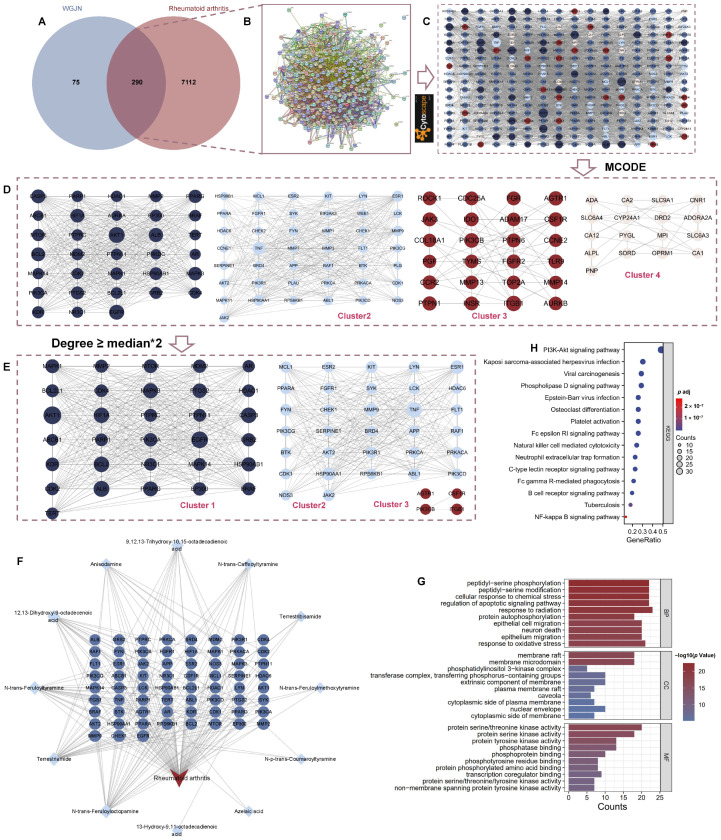
Network pharmacology analysis of Wugen San (WGS)-related targets in rheumatoid arthritis (RA). (**A**) Venn diagram showing overlapping targets between WGS-related targets and RA-associated targets. (**B**) Protein–protein interaction (PPI) network of the overlapping targets. (**C**) Visualization of the PPI network through Cytoscape software. (**D**) MCODE-identified clusters (Cluster 1–4) from the PPI network. (**E**) Further filtered PPI sub-networks (Cluster 1–3) with “degree ≥ median*2”. (**F**) Network of WGS-related active components and their corresponding RA-associated targets, with key ingredients highlighted in light blue diamonds. (**G**) GO functional enrichment analysis (BP: biological process; CC: cellular component; MF: molecular function) of the target genes. (**H**) KEGG pathway enrichment analysis. RA: rheumatoid arthritis; PPI: protein–protein interaction; MCODE: Molecular Complex Detection; KEGG: Kyoto Encyclopedia of Genes and Genomes; GO: Gene Ontology.

To clarify the action mechanism of WGS in treating RA, we systematically explored the key targets and pathways via network pharmacology. First, we retrieved the SwissADME and Swiss Target Prediction databases to collect ADME (absorption, distribution, metabolism, excretion) information and related target data of WGS active components. After removing active components that did not meet ADME criteria and excluding duplicate target information, we obtained 18 active components and 365 related targets. Meanwhile, databases such as Genecards, CTD, and DisGeNET provided 4230, 4030, and 2723 RA-associated targets, respectively. By utilizing the Venny online platform to analyze the intersection of drug-related and disease targets, 290 targets were identified to be directly linked to both the drug and the disease (Figure 4A).

To understand the interactions among these intersection targets, we constructed a PPI network. Leveraging STRING and Cytoscape (version 3.9.0) software, a PPI network was built for 290 intersection targets, containing 286 nodes and 4008 edges, with an average node degree of 28.028 (Figure 4B,C). Subsequently, the MCODE plugin was used for clustering the PPI network (k = 4), resulting in four cluster networks (Figure 4D). To identify core cluster genes, genes with a degree value ≥ twice the median in each cluster network were selected as MCODE genes; a total of 67 targets, including AKT1, TNF, EGFR, ALB, CASP3, SYK, and PTPRC, were screened out; these are considered key genes for WGS in treating RA (Figure 4E).

A “WGS components–targets–disease” network (Figure 4F) was created using Cytoscape software (version 3.9.0) to further investigate potential connections among core components of WGS, their targets, and RA. The network included 80 nodes (12 active component nodes, 67 target nodes, and one disease node) and 210 edges. Analysis of network topology parameters with the Network Analyzer plugin revealed an average of 5.250 adjacent nodes, a network heterogeneity of 1.618, a network density of 0.066, and a network centrality of 0.802. Nodes with higher degree values in the network were considered core nodes. The top-ranked active components by degree included N-trans-Feruloyloctopamine (degree = 26), Terrestriamide (degree = 24), N-trans-Feruloyltyramine (degree = 23), 12,13-Dihydroxy-9-octadecenoic acid (degree = 19), and Anisodamine (degree = 14) (Table S3). These results suggest that practical components of WGS for RA may act as central hubs with multiple action points and strong interactions in the network. Moreover, the “multi-component acting on multiple targets” characteristic was observed, indicating that WGS treats RA through multi-component and multi-target joint regulation.

To understand the biological functions of target proteins, GO functional enrichment and KEGG pathway analyses were conducted using R software (Version: 4.4.2). By applying the “Cluster Profiler” R package to 67 MCODE targets associated with RA, a total of 2516 GO terms were identified. Within these terms, 2253 were classified under the BP category, predominantly focusing on processes like peptidyl–serine phosphorylation, cellular response to chemical stress, and regulation of the apoptotic signaling pathway. Additionally, 162 terms were categorized under the MF category, including activities such as protein serine/threonine kinase activity, and 101 terms were placed in the CC category, involving structures like membrane raft and the phosphatidylinositol 3-kinase complex (Figure 4G). This suggests that potential targets play a role in biological processes linked to RA, such as inflammatory responses and cell cycles, indicating that WGS may have therapeutic effects on RA through multiple biological pathways.

Using the “Cluster Profiler” package in R, we performed KEGG pathway enrichment analysis on the 67 MCODE targets. A total of 161 signaling pathways were identified, with the PI3K-Akt signaling pathway, NF-κB signaling pathway, osteoclast differentiation, and B cell receptor signaling pathway being the main pathways (Figure 4H). This analysis revealed that targets of WGS active components are closely related to multiple pathways, and the therapeutic effect of WGS on RA features “multi-component, multi-target, and multi-pathway” characteristics, exerting multi-point actions at different levels through interrelated and synergistic multi-layer regulation.

### 2.5. Gene Expression Profiling and Machine Learning-Based Diagnostic Model for RA Identify Core Genes Associated with WGS

A systematic bioinformatic investigation was carried out to see how genes are expressed in RA. First, we searched the GEO database for “RA” and then extracted the data sets based on type and category. Finally, we selected GSE77298 and GSE89408. These datasets included 35 standard samples and 168 RA samples. Raw transcriptomic data from the RA and control groups in the GEO dataset were integrated after removing batch effects, followed by normalization. The corrected data showed a significant reduction in batch effects (Figure S1).

**Figure 5:**
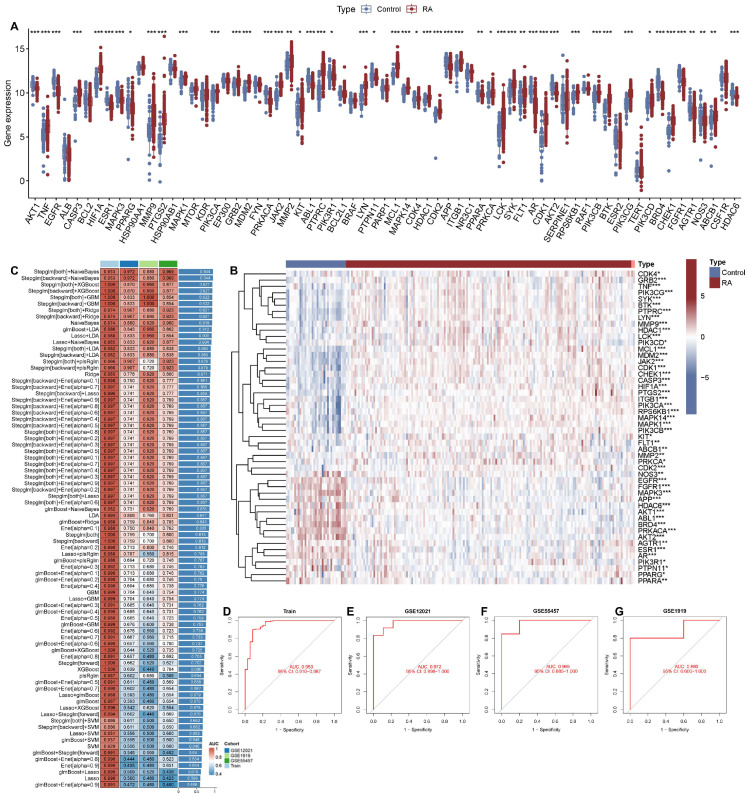
Bioinformatic analysis of gene expression profiles in rheumatoid arthritis (RA) and control groups. (**A**) Box plot showing gene expression differences between RA and control samples. Red dots represent the RA group; blue dots represent the control group. (**B**) Heatmap of differentially expressed genes (DEGs) between RA and control groups, with red indicating high expression and blue indicating low expression. (**C**) The diagnostic performance of DEGs was evaluated using a combination of 113 machine learning algorithms assessed by 10-fold cross-validation. (**D**–**G**) Receiver operating characteristic (ROC) curves for evaluating the diagnostic value of gene signatures in different RA-related datasets (GSE12021, GSE1919, GSE63084, GSE55235), with area under the curve (AUC) values indicated. RA: rheumatoid arthritis; DEGs: differentially expressed genes; GO: Gene Ontology; KEGG: Kyoto Encyclopedia of Genes and Genomes; ROC: receiver operating characteristic; AUC: area under the curve. *** *p* < 0.001, ** *p* < 0.01, * *p* < 0.05 vs. Control.

Through network pharmacology analysis, a total of 67 MCODE genes in WGS were identified (Table S4). Differential analysis of the merged GSE77298 and GSE89408 datasets identified 50 core genes (e.g., AKT1, TNF, EGFR, CASP3, HIF1A, SYK, PTPRC), which were validated in human samples, ensuring higher accuracy and clinical relevance. Among these, 17 genes (e.g., ABL1, BRD4, AGTR1, NOS3, HDAC6) were highly expressed in the normal group, while 33 genes (e.g., CHEK1, SYK, PTPRC) were upregulated in the RA group (Figure 5A,B).

To further confirm the core targets, we utilized 12 machine learning algorithms in a 10-fold cross-validation to create a robust diagnostic model based on the 50 core genes. This analysis was carried out on the training dataset and three external validation datasets (Figure 5C). By combining the Stepglm[both] and NaiveBayes algorithms, we developed a final model with optimal performance. The Stepglm[both] algorithm identified 11 key genes (ABL1, AKT2, BRD4, CHEK1, FLT1, HDAC6, MAPK3, NOS3, PIK3CB, PTPRC, SYK), while NaiveBayes filtered out the most reliable model. ROC curves of our diagnostic model (Figure 5D–G) showed high predictive efficacy for the training set (AUC = 0.953) and three validation sets (GSE1919, AUC = 0.880; GSE12021, AUC = 0.972; GSE55457, AUC = 0.969). These results indicated excellent consistency between predicted probabilities and actual clinical outcomes, confirming robust calibration performance.

### 2.6. Expression Analysis and Nomogram Model Construction of Key Genes for RA Risk Prediction

**Figure 6:**
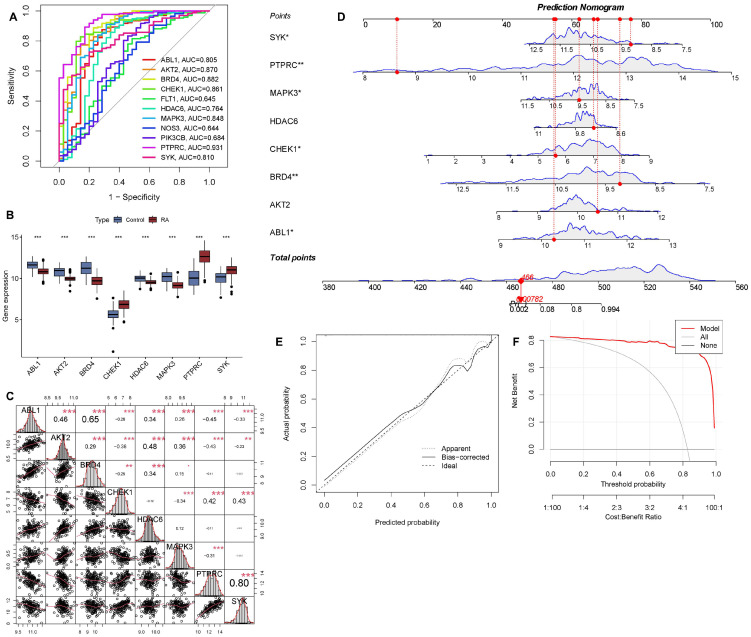
Diagnostic model construction and validation for rheumatoid arthritis (RA) based on key genes. (**A**) Receiver operating characteristic (ROC) curves of candidate genes (ABL1, AKT2, BRD4, CHEK1, HDAC6, MAPK3, NOS3, PIK3CB, PTPRC, SYK) for distinguishing RA from control samples, with area under the curve (AUC) values indicated. (**B**) Box plots showing the expression levels of the candidate genes in control and RA groups. (**C**) Correlation matrix and scatter plots of the candidate genes, with Pearson correlation coefficients (r) and significance levels (* *p* < 0.05, ** *p* < 0.01, *** *p* < 0.001) displayed. (**D**) Nomogram for predicting RA risk, integrating the expression levels of key genes. The total points correspond to the expected probability of RA. (**E**) Calibration curves for evaluating the agreement between predicted and actual RA probabilities, including apparent and bias-corrected models. (**F**) Decision curve analysis (DCA) to assess the clinical utility of the RA prediction model, comparing net benefit across different threshold probabilities. RA: rheumatoid arthritis; ROC: receiver operating characteristic; AUC: area under the curve; DCA: decision curve analysis.

To further evaluate the predictive efficiency of the machine learning model, we analyzed the expression levels and ROC curves of 11 key genes to exclude those with poor evaluation performance. ROC curve analysis revealed that 8 out of the 11 key genes (ABL1, AKT2, BRD4, CHEK1, HDAC6, MAPK3, PTPRC, SYK) exhibited good performance, with AUC values all exceeding 0.80 (Figure 6A). Subsequently, we conducted expression level and correlation analyses on these eight key genes. Results showed significant differences in the expression of all eight key genes, among which CHEK1, PTPRC, and SYK were significantly upregulated in RA samples (Figure 6B). Correlation analysis indicated strong correlations among the eight key genes, with a notably positive correlation between PTPRC and SYK (cor = 0.8) (Figure 6C). We ultimately developed a nomogram to evaluate the risk in 168 patients with RA (Figure 6D). To evaluate the predictive accuracy of the nomogram model, calibration curves and decision curve analysis (DCA) were utilized. The calibration curve indicated minimal discrepancy between the actual risk of RA samples and the predicted risk (Figure 6E). DCA confirmed that our nomogram was highly accurate and could serve as a reference for clinical decision-making (Figure 6F).

### 2.7. Immune Cell Infiltration and Key Pathways in RA

**Figure 7:**
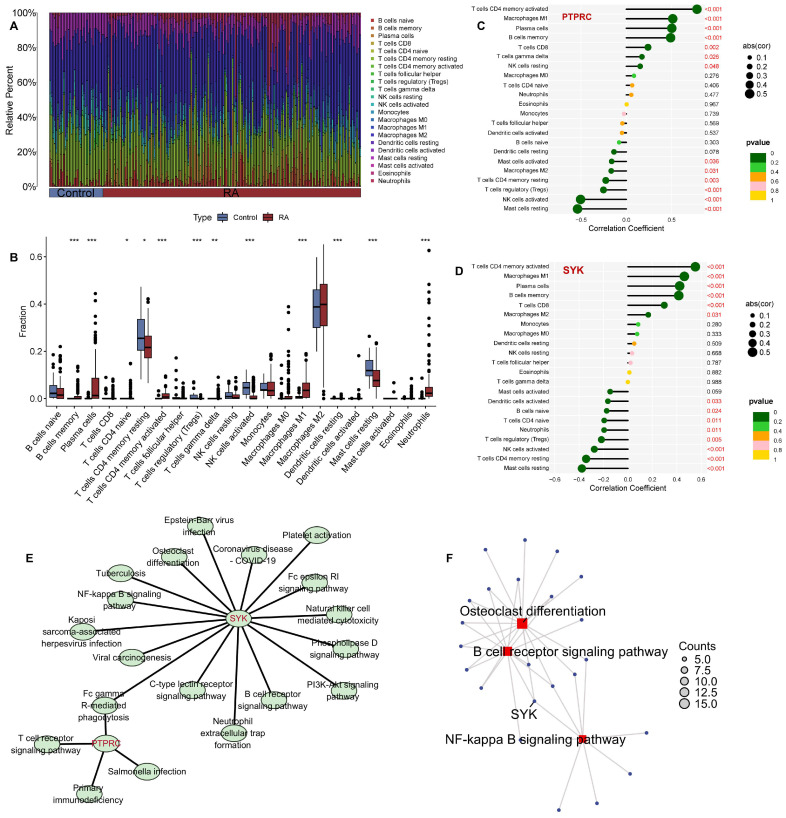
Analysis of immune cell infiltration and key gene (SYK)-related pathways in rheumatoid arthritis (RA). (**A**) Heatmap of immune cell infiltration levels in control and RA samples, with different immune cell types color-coded. (**B**) Box plots showing the fraction of various immune cell populations in control and RA groups (* *p* < 0.05, ** *p* < 0.01, *** *p* < 0.001). (**C**) Correlation analysis between PTPRC expression and immune cell infiltration levels in RA, with correlation coefficients and *p*-values indicated. (**D**) Correlation analysis between SYK expression and immune cell infiltration levels in RA, with correlation coefficients and *p*-values indicated. (**E**) Pathway enrichment analysis of SYK-related and PTPRC-related signaling pathways, visualized as a network. (**F**) Key pathways (osteoclast differentiation, B cell receptor signaling pathway, NF-kappa B signaling pathway) enriched for SYK, with node size representing gene count. RA: rheumatoid arthritis; SYK: spleen tyrosine kinase; PTPRC: protein tyrosine phosphatase receptor type C.

It is common knowledge that the development of RA is extremely complex, with the immune system being a key player in the disease. As such, we utilized the CIBERSORT algorithm to examine variations in the immune environment among RA and healthy control groups. Immune cell infiltration analysis revealed altered immune cell populations in RA. Heatmaps (Figure 7A) and box plots (Figure 7B) indicated increased infiltration of pro-inflammatory cells in RA. Notably, this altered immune infiltration was closely correlated with the expression of PTPRC and, especially, SYK. Correlation analysis showed that SYK expression was significantly correlated with the infiltration level of multiple immune cell subsets; this further illustrates the critical role of SYK in regulating the immune microenvironment in RA (Figure 7C,D). Given the importance of SYK, we further combined the pathway enrichment results of network pharmacology to visualize the related pathways of SYK and PTPRC. We found that 16 pathways were related to SYK, which was significantly more than that of PTPRC (four pathways) (Figure 7E). Further analysis revealed that SYK-related pathways, including osteoclast differentiation, the NF-κB signaling pathway and the B cell receptor signaling pathway, play a crucial role in the pathogenesis of RA (Figure 7F). The existing literature reveals that these pathways are critical for immune activation and joint destruction in RA. Therefore, we speculate that targeting SYK-related pathways may be a key mechanism for the treatment of RA by WGS.

### 2.8. WGS Regulates Key Proteins in Joint Tissues

**Figure 8:**
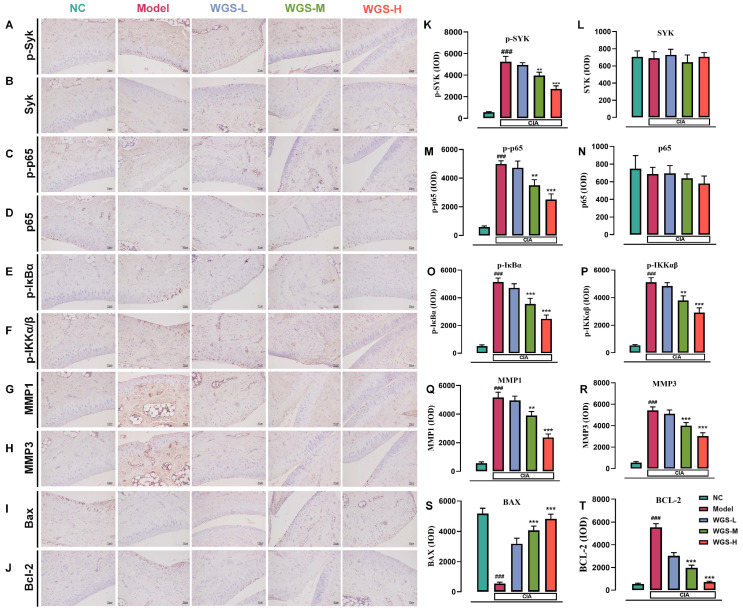
Immunohistochemical analysis of key proteins in joint tissues across different groups. (**A**–**J**) Representative immunohistochemical staining images for p-SYK (**A**), SYK (**B**), p-p65 (**C**), p65 (**D**), p-IκBα (**E**), p-IKKα/β (**F**), MMP1 (**G**), MMP3 (**H**), Bax (**I**), and Bcl-2 (**J**) (scale bar = 50 μM). (**K**–**T**) Quantitative analysis of the integrated optical density (IOD) for the respective proteins: p-SYK (**K**), SYK (**L**), p-p65 (**M**), p65 (**N**), p-IκBα (**O**), p-IKKα/β (**P**), MMP1 (**Q**), MMP3 (**R**), Bax (**S**), and Bcl-2 (**T**). NC: normal control; Model: CIA model control; WGS-L: low-dose Wugen San; WGS-M: medium-dose Wugen San; WGS-H: high-dose Wugen San. ^###^ *p* < 0.001 vs. NC; *** *p* < 0.001, ** *p* < 0.01 vs. Model.

Immunohistochemical analysis of joint tissues revealed that WGS treatment modulates the expression of key proteins involved in RA pathogenesis (Figure 8). Representative staining images showed that phosphorylated SYK (p-SYK) and phosphorylated components of the NF-κB pathway (p-p65, p-IκBα, p-IKKα/β) were significantly upregulated in the CIA model group compared to NC, while WGS administration, particularly at medium and high doses, reduced their expression in a dose-dependent manner (Figure 8A–F). Quantitative analysis confirmed these trends: p-SYK, p-p65, p-IκBα, and p-IKKα/β levels were markedly lower in WGS-M and WGS-H groups relative to the model group, whereas total SYK and p65 showed no significant expression (Figure 8K–P). Additionally, matrix metalloproteinases MMP1 and MMP3, which mediate tissue degradation, were elevated in the model group but downregulated by WGS treatment (Figure 8G,H,Q,R). For apoptosis-related proteins, the pro-apoptotic Bax was decreased in the model group, while the anti-apoptotic Bcl-2 was increased. Compared with the model group, WGS reversed these changes, with WGS-M and WGS-H significantly decreasing Bcl-2 and increasing Bax, suggesting a role in promoting joint cell apoptosis (Figure 8I,J,S,T). These findings indicate that WGS exerts therapeutic effects by suppressing NF-κB signaling, reducing matrix degradation, and regulating apoptosis in joint tissues.

## 3. Discussion

RA is a global systemic autoimmune disease with a significant incidence rate in worldwide [16]. Although traditional therapies are effective, they still have limitations such as obvious side effects, and new treatment options need to be explored urgently. Traditional Chinese medicine (TCM) and its preparations have become promising alternative therapies due to their multi-target effects and low toxicity [17,18]. In the present study, we comprehensively examined the therapeutic benefits and mechanisms of WGS in a rat model of RA, supported by analysis of its chemical composition, network pharmacology, bioinformatics, machine learning, and in vivo animal experiments. Overall, the findings show that WGS effectively relieves RA symptoms through regulation of multiple targets and pathways, achieving similar results to MTX in various important measures.

The therapeutic potential of WGS in RA was first validated in vivo using the CIA model. The dose-dependent reduction in arthritis scores, paw swelling, and recovery of body weight in WGS-treated rats directly indicate that WGS mitigates the clinical manifestations of RA, consistent with the anti-inflammatory effects of MTX. These phenotypic improvements align with findings from studies on other natural products or TCM formulas such as Ershiwuwei Lvxue Pill [18] and gastrodin [19], which also exhibit dose-dependent attenuation of arthritis severity in CIA models. The balanced regulation of cytokines by WGS, downregulating pro-inflammatory factors (e.g., TNF-α) and upregulating anti-inflammatory cytokines (e.g., IL-4), is particularly significant. Pro-inflammatory cytokines like TNF-α and IL-6 are well-established drivers of synovial inflammation and joint destruction in RA, and their inhibition is a cornerstone of RA therapy (e.g., anti-TNF biologics) [20]. WGS’s ability to modulate this cytokine network mirrors the effects of MTX, which suppresses TNF-α and IL-6 production, but extends to enhancing anti-inflammatory pathways, a feature shared with some herbal formulations like Fengshi gutong capsules [21]. Histological analyses further confirmed WGS’s protective role in joint structure: preservation of cartilage integrity, reduced osteoclast numbers (TRAP staining), and enhanced osteoblast activity (ALP staining). These findings align with existing studies showing that natural compounds or TCM formulas such as swertiamarin [22], salidroside [23] and Guizhi Shaoyao Zhimu granules [24] inhibit osteoclastogenesis and promote osteoblast function in RA models. Recently, *Toddalia asiatica* (L.) Lam. was also reported to ameliorate joint inflammation in CIA rats by reducing serum pro-inflammatory cytokines and modulating the Bax/Bcl-2 ratio to induce synovial cell apoptosis, which further supports the therapeutic paradigm of TCM-based RA intervention through multi-mechanistic regulation [25]. Notably, the inhibitory effect of WGS on MMP1 and MMP3 expression is consistent with reports that MMPs inhibition is critical for preserving cartilage integrity in RA [26].

The therapeutic benefits of WGS result from the alteration in numerous molecular and cellular pathways. In total, 67 core targets (e.g., AKT1, TNF, SYK) and key pathways (PI3K-Akt, NF-κB, osteoclast differentiation) associated with RA were obtained, which involved immune activation, inflammation, and bone metabolism disorders in RA. These pathways are also targeted by conventional RA drugs: for example, MTX inhibits NF-κB activation [27], and Janus kinase (JAK) inhibitors block cytokine signaling downstream of PI3K-Akt [28]. Network pharmacology has been widely employed to elucidate the molecular basis of TCM in RA treatment. For instance, Duhuo Jisheng pill was found to alleviate CIA via the PI3K/AKT/NF-κB signaling pathway, reducing inflammatory cytokines such as IL-6, TNF-α and IL-17A while upregulating IL-10, mirroring the multi-pathway regulation observed with WGS [29]. Similarly, Qianghuo Erhuang Decoction was shown to interfere with RA through PI3K-Akt, IL-17, TNF and HIF-1 signaling pathways, underscoring the commonality of PI3K-Akt and NF-κB pathways as key nodes for TCM action in RA [30]. Based on bioinformatics analysis, SYK is an important regulator identified, whose expression was significantly correlated with infiltration of pro-inflammatory macrophages and T cells. Furthermore, SYK was significantly involved in 16 RA-related pathways. SYK is a key kinase in B cell receptor (BCR) and Fc receptor signaling, playing an important role in the activation of immune cells and inflammatory reactions due to its signaling transducing capabilities that induce the generation of pro-inflammatory mediators. In RA, abnormal activation of SYK contributes to the atypical proliferation of synovial cells and increased infiltration of inflammatory cells, both of which characterize the pathology of RA. This is consistent with clinical evidence that SYK inhibitors (e.g., fostamatinib) reduce RA disease activity by blocking B cell and macrophage activation [31,32,33]. A recent review comprehensively summarized the emerging roles of SYK, PI3K/AKT, and GM-CSF signaling pathways in RA, emphasizing the anti-rheumatoid effects of inhibitors targeting these pathways and highlighting SYK as a promising therapeutic target for RA intervention [34]. Immunohistochemical results confirmed that WGS suppresses phosphorylated SYK (p-SYK) and key components of the NF-κB pathway (p-p65, p-IκBα, p-IKKα/β). It is noteworthy that NF-κB activation in particular is an important driver of synovial inflammation and bone destruction in RA. Inhibition of this enzyme could significantly limit the invasiveness of synovial fibroblasts and reduce their cartilage degradation ability [35]. By downregulating p-SYK, WGS likely impedes NF-κB-mediated cytokine production, forming a key mechanistic link between its suppression of immune cell activation and attenuation of joint inflammation. Additionally, WGS promoted apoptosis of pathogenic cells by upregulating Bax and downregulating Bcl-2. In RA, cells within the synovium likely evade apoptosis due to high expression of Bcl-2 [36]. Through the modulation of the Bax/Bcl-2 axis, WGS could selectively remove these pathogenic cells and lessen joint inflammation and pannus growth. This pro-apoptotic effect aligns with recent findings on other TCM-derived agents: Xuetongsu, a triterpenoid compound from Xuetong, was shown to induce RAFLS apoptosis via the Bcl-2/Bax/Caspase-3 signaling pathway, attenuating synovial hyperplasia in arthritic rats [37].

**Figure 9:**
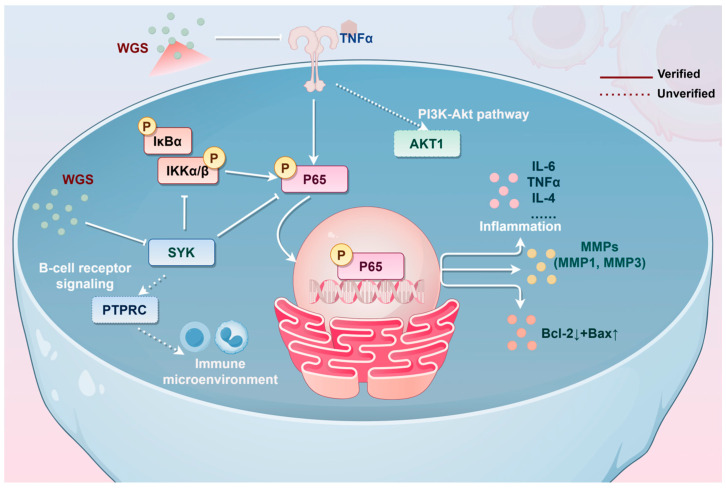
Mechanism diagram of Wugen San (WGS) against rheumatoid arthritis (RA). This diagram not only includes the validated targets and mechanisms, but also lists other potential targets and pathways predicted and verified through bioinformatics, network pharmacology, machine learning and other methods, providing a basis for further comprehensively revealing the anti-RA mechanism of WGS in the future. Wugen San (WGS); RA: rheumatoid arthritis.

Collectively, WGS exerts therapeutic effects on RA through a “multi-component, multi-target, and multi-pathway” approach. Specifically, WGS has the ability to relieve arthritis symptoms and joint damage in CIA rats in a way that is dependent on the dosage, with high-dose WGS being just as effective as MTX. It helps to manage the levels of pro-inflammatory and anti-inflammatory cytokines by reducing TNF-α, IL-6, IL-1β, and IL-17 while increasing IL-4 and IL-10 levels. Additionally, WGS inhibits osteoclast activity, promotes osteoblast function, and reduces matrix degradation by downregulating MMPs. It targets core genes (especially SYK) and suppresses the NF-κB signaling pathway to suppress inflammation. WGS also modulates the Bax/Bcl-2 axis to induce abnormal cell apoptosis in inflamed joints (Figure 9).

Several limitations of this study should be acknowledged. First, while 84 compounds were identified in WGS, the specific active components responsible for SYK and NF-κB inhibition remain unknown. Future studies should isolate and validate individual constituents, such as N-trans-Feruloyloctopamine and Terrestriamide, for their effects on these targets using molecular docking, surface plasmon resonance (SPR), and cellular assays. Second, although the CIA model is widely used, it does not fully recapitulate the autoantibody profile of human RA. Validation in human RA synovial cells or patient-derived organoid models would further confirm clinical applicability. Third, the potential of WGS to modulate adaptive immune cells (e.g., Tregs, B cells) that are important for RA pathogenesis was not explored in this study. Integrating bioinformatics and network pharmacology with GEO data has proven effective for identifying key targets and immune-related pathways in RA; future studies may adopt such an approach to systematically investigate WGS’s effects on adaptive immunity [38,39]. For example, a study on the “Tianyu” medicine pair combined LC-MS/MS, bioinformatics, machine learning and network pharmacology to identify core molecular targets such as MMP13 and NCF1, demonstrating that the integration of multi-omics and computational approaches can effectively uncover the complex mechanisms of TCM formulations in RA [40]. Fourth, the high dose of WGS (252 mg/kg) was approximately 1000-fold higher than the MTX dose (0.2 mg/kg), which raises important considerations for clinical translation regarding dosing and bioavailability. The dosage disparity between herbal extracts and chemical drugs in preclinical RA studies is common due to differences in purity, bioavailability and active component concentration; Ermiao San, for instance, was reported to exert therapeutic effects at doses of 0.75–4.5 g/kg in CIA and AIA rats, which is also considerably higher than typical MTX doses, suggesting that dose normalization based on active compound content rather than crude extract weight would be more meaningful for clinical translation [39]. Furthermore, the dose–response relationship observed in our study revealed that the therapeutic effects of WGS increased markedly from the low dose (63 mg/kg) to the medium dose (126 mg/kg), while the medium and high doses (252 mg/kg) showed comparable efficacy in most parameters. This suggests the presence of a dose threshold between WGS-L and WGS-M, beyond which additional benefits are limited. Quantitative analysis of the active components responsible for this threshold effect is critically important for optimizing clinical dosing strategies and should be addressed in future pharmacokinetic and pharmacodynamic studies. Additionally, while we attribute WGS’s therapeutic benefits to its “multi-component, multi-target” characteristics, an alternative possibility exists: a single compound within WGS might act as an agonist on one target while serving as an antagonist on another, thereby producing synergistic effects through target crosstalk rather than purely through multiple distinct components. Future studies isolating individual constituents (e.g., N-trans-Feruloyloctopamine, Terrestriamide) and testing their functional selectivity across different pathways will help distinguish between these mechanistic models. Fifth, it is important to note that while our immunohistochemical results confirmed that WGS suppresses phosphorylated SYK (p-SYK) and key components of the NF-κB pathway, these findings indicate correlation rather than direct causation. Direct evidence that SYK is the primary molecular target of specific WGS compounds, rather than a downstream correlate of broader anti-inflammatory effects, remains to be established through techniques such as cellular thermal shift assays (CETSAs), surface plasmon resonance (SPR), or genetic knockdown experiments.

## 4. Materials and Methods

### 4.1. Materials and Reagents

WGS (SFDA approval number: Z20023228) was obtained from Tibet Linzhi Yutuo Tibetan Medicine Co., Ltd. (Linzhi, Tibet, China). Methotrexate (MTX; SFDA approval number: H31020644) was obtained from SPH Sine Pharmaceutical Laboratories Co., Ltd. (Shanghai, China). Collagen type II was bought from MedChemExpress (#HY-NP003; Monmouth Junction, NJ, USA). Complete Freund’s adjuvant (CFA) was obtained from Sigma-Aldrich (#F5881; St. Louis, MO, USA). The antibodies used in this study were as follows: Antibodies against p-SYK (#AF3315, Affinity), SYK (#AF5008, Affinity), p65 (#AF5006, Affinity), p-p65 (#AF2006, Affinity), p-IκBα (#AF2002, Affinity), p-IKKα/β (#2697, CST), Bax (#50599-2-Ig, Proteintech), MMP1 (#10371-2-AP, Proteintech), MMP3 (17873-1-AP, Proteintech), and Bcl-2 (#26593-1-AP, Proteintech). RNA extraction kits (#EP013), 1st Strand cDNA Synthesis Kit (#EQ003), and SYBR Green PCR Master Mix (#EQ001) were obtained from ELK Biotechnology Co., Ltd. (Wuhan, China). Toluidine blue staining solution was purchased from Beijing WoKai Biotechnology Co., Ltd. (#GCDA0055; Beijing, China). Tartrate-resistant acid phosphatase (TRAP) staining kits were purchased from Servicebio (#G1050, Wuhan, China). Safranin O-Fast Green staining kits were bought from Solarbio (#G1371; Beijing, China). Hematoxylin–eosin (H&E, #C0105M) staining kits, Alkaline phosphatase (ALP, #C3206) staining kits and TUNEL apoptosis detection kits (#C1098) were obtained from Beyotime (Shanghai, China). All other reagents were of analytical grade.

### 4.2. Collagen-Induced Arthritis (CIA) Model Construction and Management

Specific pathogen-free male Sprague–Dawley (SD) rats weighing between 160 and 180 g were sourced from SPF (Beijing) Biotechnology Co., Ltd. (Beijing, China). They were kept in a controlled environment with a temperature of 22 ± 2 °C, humidity at 55 ± 5%, and a 12 h light/dark cycle. The rats had free access to food and water. The Animal Ethical and Welfare Committee at Chengdu University of Traditional Chinese Medicine approved all animal experiments (approval No. SCXK (Chuan) 2020-124), which were carried out following the Guide for the Care and Use of Laboratory Animals.

For the CIA model group, 36 SD rats were adaptively fed for 7 days, after which 30 rats were randomly selected for the primary immunization, recorded as day 0. An appropriate amount of bovine type II collagen solution (concentration: 2 mg·mL^−1^) was gradually added dropwise to an equal volume of complete Freund’s adjuvant (CFA), and thoroughly emulsified using a homogenizer in an ice bath until the emulsion did not diffuse in water within 30 s, resulting in a bovine type II collagen emulsifier with a concentration of 1 mg·mL^−1^. Each rat was injected with 0.2 mL of the bovine type II collagen emulsifier at the base of the tail. Feed and water were provided as normal. On the 7th day of the experiment, a booster immunization was performed using the same method, with each rat receiving an injection of 0.1 mL of the bovine type II collagen emulsifier at the base of the tail. Once the model was successfully established (on the 14th day), drug administration was initiated. The rats were gavaged once daily, and were sacrificed 21 days after the start of gavage administration for the detection of various indicators.

The rats were divided into six groups randomly, with six rats in each group: the NC group consisted of normal rats without CIA induction, administered distilled water by gavage; the model group included CIA rats administered distilled water by gavage; the MTX group had CIA rats administered MTX (0.2 mg/kg) by gavage once every 3 days; the WGS-L group comprised CIA rats administered WGS (63 mg/kg) by gavage daily; the WGS-M group had CIA rats administered WGS (126 mg/kg) by gavage daily; and the WGS-H group included CIA rats administered WGS (252 mg/kg) by gavage daily.

### 4.3. Evaluation of Arthritis Progression

Multiple parameters were measured to evaluate arthritis progression. The severity of arthritis was scored every three days using a 0–4 grading system. A score of 4 indicated swelling in the entire paw, including the ankle joint. The arthritis score for each rat was determined based on the standard scoring system, in which a score of maximum 4 was assigned for each joint. The total score for all four limbs of the rat formed the total arthritis score, with a maximum possible score of 16. Successful establishment of the model was considered with arthritis score ≥ 4. Additionally, the weight of the rat bodies was measured every three days during the experiment. The thickness of the hind paws was measured using a vernier caliper every three days, and the degree of swelling was calculated as the difference between the post-immunization and pre-immunization thicknesses.

### 4.4. Histopathological Analysis of Joint Tissues

After the experiment, rats were euthanized, after which their hind ankle joints were dissected. The joints were then fixed in 4% paraformaldehyde for 48 h, decalcified in 10% ethylenediaminetetraacetic acid (EDTA) for 4 weeks, embedded in paraffin, and sectioned at a thickness of 5 μm. These sections underwent multiple staining procedures for comprehensive histopathological analysis. H&E staining was employed to evaluate synovial hyperplasia, cartilage erosion, inflammatory cell infiltration, and bone destruction; Toluidine blue and Safranin O-Fast Green staining were used to assess proteoglycan content and cartilage integrity; TRAP staining served to identify osteoclasts (TRAP-positive multinucleated cells); and ALP staining was carried out to evaluate osteoblast activity (ALP-positive cells). Finally, two independent pathologists analyzed the histopathological changes according to established criteria using a blind method.

### 4.5. TUNEL Assay for Apoptotic Cells in Joint Tissues

Apoptosis in joint tissues was assessed using the TUNEL assay as per the manufacturer’s guidelines. Briefly, paraffin sections were processed by deparaffinizing, rehydrating, and treating with proteinase K. The sections were then exposed to the TUNEL reaction mixture at 37 °C for 1 h, followed by DAPI staining to visualize nuclei. The number of TUNEL^+^/DAPI^+^ cells (apoptotic cells) was quantified in three random fields per section under a fluorescence microscope (×200). The apoptosis rate was determined by calculating the ratio of TUNEL^+^ cells to total DAPI^+^ cells and multiplying by 100%.

### 4.6. qRT-PCR for Cytokine mRNA Expression

Joint tissues were used for the extraction of total RNA with TRIzol reagent, followed by cDNA synthesis using a reverse transcription kit. Subsequently, qRT-PCR was carried out on a StepOnePlus Real-Time PCR System (Thermo Fisher Scientific, USA) using SYBR Green PCR Master Mix. The primer sequences are available in Table S1. Relative mRNA expression levels were calculated using the 2^−ΔΔCt^ method, with GAPDH serving as the internal reference gene.

### 4.7. UPLC-Q-TOF/MS Analysis of Chemical Components in WGS

#### 4.7.1. Sample Preparation

Weigh 1.0 g of WGS sample precisely, place it in a 50 mL stoppered conical flask, add 15 mL of 50% methanol, and subject it to ultrasonic treatment (300 W power, 40 kHz frequency) for 30 min. Afterward, remove it, allow it to cool, shake thoroughly, transfer 2 mL to a centrifuge tube, centrifuge at a high speed (12,000 r/min) for 5 min, and filter the resulting supernatant through a 0.22 μm filter membrane to obtain the final sample solution for UPLC-Q-TOF/MS analysis.

#### 4.7.2. UPLC-Q-TOF/MS Conditions

The separation was carried out on a Waters ACQUITY UPLC HSS T3 column (2.1 × 100 mm, 1.8 μm) at a temperature of 30 °C using the following mobile phase composition: acetonitrile (A) and 0.1% formic acid aqueous solution (B). A gradient elution program was employed with the following time intervals: 0–3 min, 0% A (100% B); 3–6 min, 0–6% A (100–94% B); 6–15 min, 6–20% A (94–80% B); 15–30 min, 20–35% A (80–65% B); 30–33 min, 35–45% A (65–55% B); 33–35 min, 45–95% A (55–5% B); 35–39 min, 95% A (5% B); 39–39.1 min, 95–0% A (5–100% B); 39.1–41 min, 0% A (100% B). The flow rate was set at 0.3 mL/min, and a 2 μL injection volume was used. Detection was performed at a wavelength of 254 nm within a range of 190–400 nm.

Mass spectrometry analysis was done in ESI-Negative/Positive ion modes with the following settings: TOF mass range of 50–1700; Ion Source Gas 1 at 50 psi; Ion Source Gas 2 at 50 psi; Curtain Gas at 35 psi; Ion Spray Voltage Floating −4500/5000 V; Ion Source Temperature at 500 °C; Declustering Potential at 100 V; Collision Energy at 10 eV. For MS/MS parameters, the settings were as follows: MS/MS mass range of 50–1250; Collision Energy at ±40 eV; Collision Energy Spread at 20 eV; Ion Release Delay at 30 ms; Ion Release Width at 15 ms.

#### 4.7.3. Data Processing and Compound Identification

Analyst TF 1.7.1 software was used for data acquisition, while Peakview 1.2 software was utilized for data processing. Initially, the mass spectrometry data was compared with the Natural Products HR-MS/MS Spectral Library 1.0 from Shanghai Standard Technology Co., Ltd. to identify compounds. The library contains actual multi-stage mass spectra of reference standards with detailed information to ensure accurate matching results. Compounds were first screened based on the score of each chromatographic peak and then confirmed using primary and secondary mass spectral information. If a compound was not found in the database, identification was done based on literature reports and mass spectral fragmentation rules.

### 4.8. Network Pharmacology Analysis

#### 4.8.1. Screening of Active Components and Prediction of Targets

The SMILES structures of components in WGS were retrieved from the PubChem database (https://pubchem.ncbi.nlm.nih.gov/ accessed on 16 August 2024 and then inputted into the SwissADME database (http://www.swissadme.ch/ accessed on 15 March 2026) for predicting ADME properties, with screening criteria set as high gastrointestinal absorption (GI absorption = “High”) and at least two out of the five druggability predictions (Lipinski, Ghose, Veber, Egan, Muegge) being “Yes”. Next, the SMILES structures of the selected active components were fed into the SwissTarget Prediction database (http://www.swisstargetprediction.ch/ accessed on 16 August 2024) to forecast their targets, with a probability score > 0.1 as the screening principle. The UniProtIDs of the target proteins were converted to Gene symbols via the ID mapping tool in the UniProt database (https://www.uniprot.org/ accessed on 16 August 2024), and any duplicate targets were eliminated to identify the active component targets of WGS.

#### 4.8.2. Collection of RA-Related Targets

RA-related genes were collected from three databases of disease genes, including CTD (https://ctdbase.org/ accessed on 16 August 2024), GeneCards (https://www.genecards.org/ accessed on 16 August 2024), and DisGeNET (https://www.disgenet.org accessed on 16 August 2024), using “rheumatoid arthritis” as the search term. The data obtained from these databases were structured and assembled for the RA-related targets.

#### 4.8.3. Construction of PPI Network

The Venny online tool (https://bioinfogp.cnb.csic.es/tools/venny/ accessed on 16 August 2024) was utilized to identify the overlapping targets between WGS and RA, which were considered effective therapeutic targets for treating RA. These shared targets were inputted into the STRING database (https://cn.string-db.org/ accessed on 16 August 2024) to create a protein–protein interaction (PPI) network with an interaction score cutoff of ≥0.4. The PPI network diagram and TSV file were downloaded for further analysis. Cytoscape software version 3.9.0 was employed to visualize the PPI network and construct the multi-dimensional “WGS-RA” network. Cluster analysis of the PPI network was conducted using the MCODE plugin to identify specific targets of WGS in the treatment of RA.

#### 4.8.4. “Component–Target–Disease” Network Construction

The construction of a network named “WGS component–target–RA” was completed using Cytoscape 3.9.0. By utilizing the Network Analyzer plugin, the topology parameters (Degree, Betweenness Centrality, Closeness Centrality) were analyzed, and core components were identified based on their degree values.

#### 4.8.5. GO and KEGG Enrichment Analyses

Functional enrichment analysis, including biological process (BP), molecular function (MF), and cellular component (CC) analysis, and KEGG pathway enrichment analyses of MCODE targets were conducted in R software. The screening criteria were established as q value < 0.05, and the enrichment results were arranged in decreasing order according to *p*-value to identify significantly different enrichment outcomes. The enriched GO biological functions and the top 20 KEGG signaling pathways were output separately.

### 4.9. Gene Expression Profiling and Machine Learning-Based Diagnostic Model

#### 4.9.1. Data Collection and Preprocessing

Datasets related to RA gene expression were obtained from the GEO database, with the filters set as “array expression profiles” and “Homo sapiens”. The training datasets included GSE77298 containing 16 RA patient samples and seven healthy control samples, and GSE89408 consisting of 152 RA patient samples and 28 healthy control samples. The validation datasets included GSE1919 with 5 RA patient samples and five healthy control samples, GSE12021 comprising 12 RA patient samples and nine healthy control samples, and GSE55457 including 13 RA patient samples and 10 healthy control samples. All data were publicly available from GEO, thus no ethical approval or informed consent was required. The training datasets (GSE77298 and GSE89408) were merged, followed by batch effect removal and normalization. Perl scripts were used for gene symbol annotation and data correction to obtain the expression levels of intersection genes (from network pharmacology) in normal and RA samples.

#### 4.9.2. Identification of Core Genes

R packages were used to analyze MCODE genes. The analysis used limma, heatmap, and ggpubr R packages to analyze MCODE genes in healthy controls and RA. We selected genes with *p* < 0.05 as core genes, and we visualized differentially expressed MCODE genes using boxplots and heatmaps.

#### 4.9.3. Machine Learning-Based Diagnostic Model Construction, Validation, and Nomogram Development

Twelve machine learning algorithms, including as Lasso, GBM, NaiveBayes, LDA, and SVM, were utilized to construct diagnostic models. By testing 113 different combinations of these algorithms through a 10-fold cross-validation approach with the training datasets (GSE77298 and GSE89408), variable selection and model development were carried out. The models were then externally validated with GSE1919, GSE12021, and GSE55457 datasets. The model showing the highest average AUC across both training and test sets was chosen as the most optimal, and ROC curves were plotted to evaluate its diagnostic performance. Finally, utilizing the key genes and their expression levels in normal and RA groups, a nomogram model was created, where each predictor was given a score corresponding to their importance, and the total score was represented as “Points”.

### 4.10. Immune Cell Infiltration Analysis

The immune cell infiltration of RA samples was analyzed by utilizing the “CIBERSORT” package in R software with 1000 permutations. Correlations between significant genes (SYK, PTPRC) and infiltration levels of immunity cells were examined using Spearman’s correlation coefficient.

### 4.11. Immunohistochemical (IHC) Analysis of Key Proteins

After deparaffinization and rehydration, paraffin sections of joint tissues underwent antigen retrieval before being treated with primary antibodies against various proteins including p-SYK, SYK, p-p65, p65, p-IκBα, p-IKKα/β, MMP1, MMP3, Bax, and Bcl-2 overnight at 4 °C. The sections were then exposed to HRP-conjugated secondary antibodies and visualized with DAB substrate, followed by counter-staining with hematoxylin. The integrated optical density (IOD) of positive staining was assessed using Image-Pro Plus 6.0 software in five random fields for each section.

### 4.12. Statistical Analysis

All experiments were performed with at least three independent biological replicates. All data are presented as mean ± standard deviation (SD). Statistical analysis was conducted using GraphPad Prism 10.4.2 and R 4.2.0. Normality and homogeneity of variance were verified before hypothesis testing. One-way analysis of variance (ANOVA) followed by Tukey’s post hoc test was used to compare differences between multiple groups. *p* < 0.05 was considered to be statistically significant.

## 5. Conclusions

This study integrated chemical component identification, network pharmacology, bioinformatics, machine learning and in vivo animal experiments to systematically investigate the therapeutic effects and mechanisms of WGS in treating RA. Through systematic evaluation in a CIA rat model, we demonstrated that WGS significantly attenuates disease progression, reduces pro-inflammatory cytokines, and enhances anti-inflammatory mediators in a dose-dependent manner, with high-dose efficacy matching MTX. Mechanistically, WGS preserves joint architecture by suppressing osteoclastogenesis, promoting osteoblast activity, and inducing apoptosis of pathological cells, while UPLC-Q-TOF/MS characterization identified 84 bioactive compounds. Network pharmacology and machine learning-driven biomarker discovery pinpointed SYK as a pivotal target, with subsequent validation confirming that WGS downregulates SYK/NF-κB signaling and MMPs in arthritic joints. These results collectively establish WGS as a multi-component, multi-target agent modulating critical RA pathways (NF-κB, osteoclast differentiation, etc.). This study provides a pharmacological basis for repurposing WGS for RA treatment and identifies SYK-centered networks as potential therapeutic nodes. Future efforts should focus on isolating and validating individual active compounds, evaluating WGS efficacy in human clinical trials, and exploring SYK-targeting phytocompounds for precision medicine in autoimmune arthritis.

## Data Availability

The original contributions presented in this study are included in the article/Supplementary Material. Further inquiries can be directed to the corresponding authors.

## Supplementary Materials

The following supporting information can be downloaded at: https://www.mdpi.com/article/10.3390/ph19050718/s1, Figure S1: Normalization of the GSE77298 and GSE89408 datasets; Table S1: Primer sequence information; Table S2: Identification of chemical components of Wugen San (WGS) by using UPLC-Q-Exactive Orbitrap-MS [41,42,43,44,45,46,47,48,49,50,51,52,53,54,55,56,57,58,59,60,61,62,63,64,65,66,67,68,69,70,71,72,73,74]; Table S3: Top-ranked Degree value active ingredients; Table S4. Network pharmacology analysis identified 67 MCODE genes in WGS.

## Abbreviations

The following abbreviations are used in this manuscript:

RA	Rheumatoid arthritis
WGS	Wugen San
CIA	Collagen-induced arthritis
MTX	Methotrexate
TCM	Traditional Chinese medicine
ALP	Alkaline phosphatase
TRAP	Tartrate-resistant acid phosphatase
SD	Sprague–Dawley
CFA	Complete Freund’s adjuvant
EDTA	Ethylenediaminetetraacetic acid
BP	Biological process
MF	Molecular function
CC	Cellular component
PPI	Protein–protein interaction
IOD	Integrated optical density
DCA	Decision curve analysis
