# Supplementary material for: Revealing the Pharmacological Mechanism of Tibetan Medicine Wugeng San in Treating Rheumatoid Arthritis Through an Integrated Strategy of Chemical Composition Analysis, Network Pharmacology, Machine Learning, and In Vivo Experiments
Source: Pharmaceuticals (Basel). 2026 Apr 30;19(5):718. doi: 10.3390/ph19050718 (PMC13209509; doi:10.3390/ph19050718)
Supplement: Supplementary file 1 [file pharmaceuticals-19-00718-s001.zip › pharmaceuticals-4259751-supplementary.pdf]

Supplementary Materials

For

Revealing the Pharmacological Mechanism of Tibetan Medicine Wugeng San in treating Rheumatoid Arthritis through an Integrated Strategy of Chemical Composition Analysis, Network Pharmacology, Machine Learning, and in vivo Experiments

Figure S1. Normalization of the GSE77298 and GSE89408 datasets. (A) Before normalization. (B) After normalization. (C) Before batch removal. (D) After the batch.

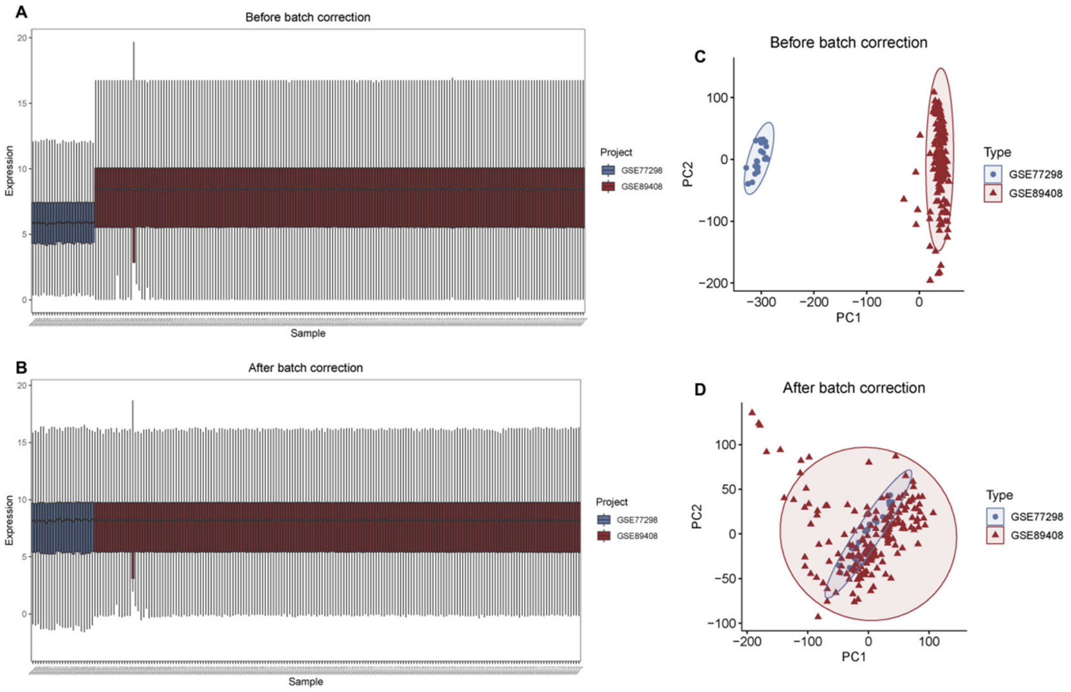

Table S1. Primer sequence information.

Gene		Primer sequence（5`-3`）	Product length
R-GAPDH	Forward	GCCAAGGTCATCCATGACAAC	152
	Reverse	GTGGATGCAGGGATGATGTTC	
R-TNF-a	Forward	CACCACGCTCTTCTGTCTACTG	147
	Reverse	GCTACGGGCTTGTCACTCG	
R-IL-6	Forward	GCCAGAGTCATTCAGAGCAAT	150
	Reverse	CTTGGTCCTTAGCCACTCCT	
R-IL-1β	Forward	GTGGCAGCTACCTATGTCTTGC	251
	Reverse	CCACTTGTTGGCTTATGTTCTGT	
R-IL-17	Forward	CTCAGACTACCTCAACCGTTCC	198
	Reverse	CACTTCTCAGGCTCCCTCTTC	
R-IL-4	Forward	TGTACCTCCGTGCTTGAAGAAC	175
	Reverse	CTGCAAGTATTTCCCTCGTAGG	
R-IL-10	Forward	ACTGCTATGTTGCCTGCTCTTAC	199
	Reverse	CAGTAAGGAATCTGTCAGCAGTATG	

Table S2. Identification of chemical components of Wugen San (WGS) by using UPLC-Q-Exactive Orbitrap-MS.

NO.	t_R (min)	Ion mode	Actual value (*m/z*)	Theoretical value (*m/z*)	ppm	Molecular formula	Compounds	Fragment ions	Attribution	Category	Database comparison	Ref.
1	1.07	$[M+FA-H]^-$	387.1158	387.1144	3.6	$C_{12}H_{22}O_{11}$	Sucrose	387.1158, 341.1101, 179.0568, 161.0463	H, T	Saccharides	√	
2	1.42	$[M+FA-H]^-$	549.1695	549.1672	4.2	$C_{18}H_{32}O_{16}$	D-(+)-Raffinose	549.1695, 503.1639, 341.1105, 221.0672, 179.0567	H, T	Saccharides	√	
3	2.14	$[M-H]^-$	191.0204	191.0197	3.7	$C_6H_8O_7$	Citric acid	191.0204, 111.0080, 87.0083, 85.0292, 67.0185	H, T	Organic acids	√	
4	2.78	$[M+FA-H]^-$	711.2234	711.2201	4.6	$C_{24}H_{42}O_{21}$	Fungitetraose	711.2234, 665.2140, 383.1164, 341.1068	H, T	Saccharides	/	
5	3.02	$[M+FA-H]^-$	711.2234	711.2201	4.6	$C_{24}H_{42}O_{21}$	Maltotetraose	/	H, T	Saccharides	/	
6	5.79	$[M-H]^-$	827.2717	827.2674	5.2	$C_{30}H_{52}O_{26}$	Maltopentaose	827.2717, 665.2094, 545.1680, 383.1190, 179.0555	H, T	Saccharides	√	
7	6.21	$[M+H]^+$	268.1044	268.1040	1.5	$C_{10}H_{13}N_5O_4$	Adenosine	268.1044, 136.0608, 119.0336, 94.0385	X, H	Nucleosides	√	
8	6.25	$[M+FA-H]^-$	873.2771	873.2729	4.8	$C_{30}H_{52}O_{26}$	1F-Fructofuranosylnystose	/	H, T	Saccharides	√	
9	6.55	$[M+FA-H]^-$	1035.3318	1035.3257	5.9	$C_{36}H_{62}O_{31}$	Maltohexaose	1035.3318, 989.3274, 827.2756, 647.2076, 341.1097	H, T	Saccharides	/	
10	6.73	$[M+FA-H]^-$	1197.3868	1197.3785	6.9	$C_{42}H_{72}O_{36}$	1-Kestoheptaose	1197.3868, 1151.3708, 989.3227, 869.2815	H, T	Saccharides	√	
11	6.84	$[M+FA-H]^-$	1035.3319	1035.3257	6.0	$C_{36}H_{62}O_{31}$	1,1,1,1-Kestohexose	1035.3319, 989.3274, 827.2756, 665.2149, 341.1097	H, T	Saccharides	√	
12	6.90	$[M+FA-H]^-$	1197.3867	1197.3785	6.8	$C_{42}H_{72}O_{36}$	Maltoheptaose	1197.3867, 1151.3758, 989.3203, 827.2706	H, T	Saccharides	/	
13	7.77	$[M-H]^-$	283.0690	283.0687	1.1	$C_{10}H_{12}N_4O_6$	Xanthosine	283.0690, 151.0257, 108.0208	H, T	Nucleosides	√	
14	8.60	$[M-H]^-$	315.0725	315.0722	1.0	$C_{13}H_{16}O_9$	Gentisic acid 5-O-β-glucoside	315.0725, 152.0119, 108.0224	H, T	Phenolic acids	/	[1]
15	8.75	$[M+H]^+$	127.0389	127.0390	-0.8	$C_6H_6O_3$	5-Hydroxymethylfurfural	127.0389, 109.0274, 81.0319, 71.0125	H, T	Furanaldehydes	√	
16	9.06	$[M-H]^-$	329.0888	329.0878	3.0	$C_{14}H_{18}O_9$	Methyl 5-(β-D-glucopyranosyloxy)-2-hydroxybenzoate	329.0888, 167.0350, 152.0115, 123.0445, 108.0210	H	Phenolic acids	/	[2]
17	9.64	$[M+H]^+$	188.0710	188.0706	2.1	$C_{11}H_9NO_2$	3-Indoleacrylic acid	188.0710, 170.0616, 146.0607, 118.0650, 115.0536	H	Alkaloids	/	[3]
18	9.69	$[M-H]^-$	203.0832	203.0826	3.0	$C_{11}H_{12}N_2O_2$	L-Tryptophan	203.0832, 159.0929, 142.0665, 116.0501	X, J, H	Alkaloids	√	[4]
19	10.44	$[M-H]^-$	137.0249	137.0244	3.6	$C_7H_6O_3$	4-Hydroxybenzoic acid	137.0249, 108.0217, 92.0284	X	Phenolic acids	√	[5]
20	10.85	$[M+H]^+$	304.1547	304.1543	1.3	$C_{17}H_{21}NO_4$	(-)-Scopolamine	304.1547, 156.1015, 138.0908, 121.0632, 103.0543	J, H, T	Alkaloids	√	

21	11.30	$[M+H]^+$	306.1705	306.1700	1.6	$C_{17}H_{23}NO_4$	Anisodamine	306.1705, 140.1069, 122.0958, 122.0958, 103.0537	H	Alkaloids	/	
22	11.71	$[M+FA-H]^-$	531.1382	531.1355	5.1	$C_{21}H_{26}O_{13}$	Fabiatrin	531.1382, 485.1332, 191.0360, 176.0118, 148.0166	/	Coumarins	√	
23	11.76	$[M-H]^-$	353.0891	353.0878	3.7	$C_{16}H_{18}O_9$	Chlorogenic acid	353.0891, 191.0570, 173.0445, 161.0229, 127.0370	L	Phenolic acids	√	
24	11.85	$[M-H]^-$	289.0724	289.0718	2.1	$C_{15}H_{14}O_6$	(-)-Epicatechin	289.0724, 245.0804, 205.0509, 188.0476, 151.0400	J	Flavonoids	√	[6]
25	12.92	$[M+H]^+$	290.1761	290.1751	3.4	$C_{17}H_{23}NO_3$	(-)-Atropine	290.1761, 142.1230, 124.1123, 93.0697	H	Alkaloids	√	[7]
26	13.08	$[M+FA-H]^-$	431.1943	431.1923	4.6	$C_{19}H_{30}O_8$	Corchoionoside C	431.1943, 385.1845, 179.0499, 153.0955, 119.0349	H	Monoterpene glycosides	/	
27	13.46	$[M-H]^-$	289.0725	289.0718	2.4	$C_{15}H_{14}O_6$	Catechin	289.0725, 245.0818, 203.0715, 151.0392	J	Flavonoids	√	[6]
28	14.06	$[M+FA-H]^-$	461.1688	461.1665	5.0	$C_{19}H_{28}O_{10}$	Phenethyl 6-O-D-apiosyl-β-D-glucopyranoside	461.1688, 415.1607, 251.0768, 119.0341	X	Phenols	/	
29	14.83	$[M+FA-H]^-$	787.2706	787.2666	5.1	$C_{34}H_{46}O_{18}$	Syringaresinol di-O-β-D-glucoside	787.2706, 741.2705, 579.2108, 417.1582, 181.0528	X	Lignans	√	
30	15.60	$[M+H]^+$	193.0502	193.0501	0.5	$C_{10}H_8O_4$	Scopoletin	193.0502, 178.0261, 150.0312, 133.0288, 122.0359	L, X, J, H, T	Coumarins	√	
31	15.81	$[M-H]^-$	193.0512	193.0506	3.1	$C_{10}H_{10}O_4$	Ferulic acid	193.0512, 178.0257, 134.0374, 121.0309	L, J	Phenolic acids	√	
32	16.03	$[M-H]^-$	609.1498	609.1461	6.1	$C_{27}H_{30}O_{16}$	Rutin	609.1498, 300.0274, 271.0235, 255.0299	J, H	Flavonoid glycosides	√	
33	16.22	$[M+H]^+$	743.2059	743.2029	4.0	$C_{32}H_{38}O_{20}$	Quercetin 3-O-(2^G-β-D-xylopyranosylrutinoside)	743.2029, 611.1649, 449.1107, 303.0615	J	Flavonoid glycosides	/	[8]
34	16.52	$[M-H]^-$	463.0904	463.0882	4.8	$C_{21}H_{20}O_{12}$	Isoquercitrin	463.0904, 300.0276, 271.0256, 255.0296	J, H	Flavonoid glycosides	√	
35	16.79	$[M+H-H_2O]^+$	312.1217	312.1230	-4.2	$C_{18}H_{19}NO_5$	N-trans-Feruloyloctopamine	312.1192, 177.0541, 145.0284, 117.0328	J, H	Amides	√	
36	17.25	$[M+H]^+$	595.1685	595.1657	4.7	$C_{27}H_{30}O_{15}$	3,4′,5,7-Tetrahydroxy-3-[6-O-(6-deoxy-β-L-mannopyranosyl)-D-glucopyranoside]-flavone	595.1685, 449.1100, 287.0562	J	Flavonoid glycosides	/	[9]

37	17.33	$[M-H]^-$	609.1497	609.1461	5.9	$C_{27}H_{30}O_{16}$	Quercetin 7-O-rutinoside	609.1461, 300.0265, 271.0219, 255.0294	H	Flavonoid glycosides	/	
38	17.48	$[M-H]^-$	579.2111	579.2083	4.8	$C_{28}H_{36}O_{13}$	(-)-Syringaresinol 4-O-β-D-glucopyranoside	579.2111, 417.1575, 402.1364, 181.0510, 166.0269	X	Lignans	√	[10]
39	17.68	$[M-H]^-$	515.1216	515.1195	4.1	$C_{25}H_{24}O_{12}$	Isochlorogenic acid B	515.1216, 353.0870, 191.0555, 161.0257, 135.0431	L	Phenolic acids	√	
40	17.81	$[M-H]^-$	515.1218	515.1195	4.5	$C_{25}H_{24}O_{12}$	Isochlorogenic acid A	515.1218, 353.0894, 191.0574, 179.0358, 135.0457	L	Phenolic acids	√	
41	18.16	$[M-H]^-$	298.1092	298.1085	2.3	$C_{17}H_{17}NO_4$	N-trans-Caffeoyltyramine	298.1092, 178.0517, 161.0244, 135.0448	J, H	Amides	√	
42	18.18	$[M-H]^-$	187.0982	187.0976	3.2	$C_9H_{16}O_4$	Azelaic acid	187.0982, 169.0863, 125.0971, 123.0837	L, H	Organic acids	√	
43	18.45	$[M+H]^+$	679.5149	679.5078	10.4	$C_{36}H_{66}N_6O_6$	Cyclic hexaleucine	679.5149, 661.5092, 336.2334, 309.0755	H	Cyclic peptides	/	
44	19.15	$[M+H]^+$	755.4249	755.4212	4.9	$C_{39}H_{62}O_{14}$	Pallidifloside C	755.4249, 593.3714, 431.3160	H	Steroidal saponins	/	[11]
45	19.71	$[M+FA-H]^-$	981.4976	981.4912	6.5	$C_{45}H_{76}O_{20}$	Timosaponin E1	981.4976, 935.4880, 773.4361, 611.3863	T	Steroidal saponins	/	[12]
46	20.38	$[M-H]^-$	282.1143	282.1136	0.7	$C_{17}H_{17}NO_3$	N-p-trans-Coumaroyltyramine	282.1143, 162.0562, 145.0314, 119.0511	J, H	Amides	/	[13]
47	20.94	$[M+H]^+$	328.1192	328.1179	4.0	$C_{18}H_{17}NO_5$	Terrestriamide	328.1192, 177.0547, 145.0285, 117.0343	J	Amides	/	
48	21.08	$[M-H]^-$	312.1252	312.1241	3.5	$C_{18}H_{19}NO_4$	N-trans-Feruloyltyramine	312.1252, 297.1038, 190.0499, 178.0509, 148.0526	J, H	Amides	√	[14]
49	21.26	$[M-H]^-$	1051.5418	1051.5331	8.3	$C_{50}H_{84}O_{23}$	25S-Officinalisnin II	1051.5418, 919.4913, 889.4772, 757.4346	T	Steroidal saponins	/	[15]
50	21.41	$[M+FA-H]^-$	1097.5482	1097.5385	8.8	$C_{50}H_{84}O_{23}$	25-epi-Officinalisnin II	1097.5482, 1051.5374, 919.4949, 889.4856, 757.4421	T	Steroidal saponins	/	[16]
51	21.73	$[M+H]^+$	441.2031	441.2020	2.5	$C_{24}H_{28}N_2O_6$	Terrestribisamide	441.2031, 265.1573, 177.0550, 145.0291	J	Amides	/	[17]
52	21.79	$[M+FA-H]^-$	965.5043	965.4963	8.3	$C_{45}H_{76}O_{19}$	Disporoside C	965.5043, 919.4981, 757.4468, 595.3907	T	Steroidal saponins	/	[18]
53	21.94	$[M+FA-H]^-$	965.4967	965.4963	0.4	$C_{45}H_{76}O_{19}$	(25S)-Officinalisnin-I	965.4967, 919.4936, 757.4394, 595.3784	T	Steroidal saponins	/	[16]
54	22.52	$[M+H]^+$	344.1505	344.1492	3.8	$C_{19}H_{21}NO_5$	N-trans-Feruloylmethoxytyramine	344.1505, 177.0558, 145.0284, 117.0344	J, H	Amides	/	
55	22.90	$[M+FA-H]^-$	1109.5476	1109.5385	8.2	$C_{51}H_{84}O_{23}$	Protogracillin	1109.5476, 1063.5417, 901.4893, 755.4362	J	Steroidal saponins	/	[19]
56	22.94	$[M+H]^+$	1047.5435	1047.5371	6.1	$C_{51}H_{82}O_{22}$	Pseudoprotogracillin	1047.5435, 885.49069, 723.42481, 577.3779	J	Steroidal saponins	/	[20]
57	23.07	$[M+H]^+$	901.4843	901.4791	5.8	$C_{45}H_{72}O_{18}$	Neosibiricoside D	901.4843, 739.4291, 577.3757, 415.3223	H	Steroidal saponins	/	[21]

58	23.12	$[M+FA-H]^-$	1111.5629	1111.5542	7.8	$C_{51}H_{86}O_{23}$	(3β,5β,22α,25R)-26-(β-D-glucopyranosyloxy)-22-hydroxyfurostan-3-yl O-6-deoxy-α-L-mannopyranosyl-(1→4)-O-[β-D-glucopyranosyl-(1→2)]-β-D-Glucopyranoside,	1111.5629, 1065.5545, 903.5029, 757.4439, 595.3798	T	Steroidal saponins	/	[15]
59	23.29	$[M+FA-H]^-$	1093.5528	1093.5436	8.4	$C_{51}H_{84}O_{22}$	Protodioscin	1093.5528, 1047.5444, 901.4909, 755.4242, 593.3759	J, H, T	Steroidal saponins	√	
60	23.34	$[M+H]^+$	885.4893	885.4842	5.8	$C_{45}H_{72}O_{17}$	Gracillin	885.4893, 723.4342, 577.3782, 415.3232	J, H, T	Steroidal saponins	√	
61	23.42	$[M+FA-H]^-$	1081.5524	1081.5436	8.1	$C_{50}H_{84}O_{22}$	(3β,5β,25S)-26-(β-D-Glucopyranosyloxy)-22-hydroxyfurostan-3-yl O-6-deoxy-α-L-mannopyranosyl-(1→6)-O-[β-D-xylopyranosyl-(1→4)]-β-D-glucopyranoside	1081.5524, 1035.5407, 903.4968, 889.4817, 757.4397	T	Steroidal saponins	/	[22]
62	23.59	$[M-H]^-$	1035.5462	1035.5381	7.8	$C_{50}H_{84}O_{22}$	(3β,5β,22α,25S)-26-(β-D-Glucopyranosyloxy)-22-methoxyfurostan-3-yl O-β-D-xylopyranosyl-(1→2)-O-[β-D-xylopyranosyl-(1→4)]-β-D-glucopyranoside	/	T	Steroidal saponins	/	[23]
63	23.83	$[M+FA-H]^-$	947.4923	947.4857	7.0	$C_{45}H_{74}O_{18}$	Protobioside I	947.4923, 901.4835, 755.4373, 593.3963	H	Steroidal saponins	/	[24]
64	24.01	$[M+FA-H]^-$	949.5088	949.5014	7.8	$C_{45}H_{76}O_{18}$	(3β,5β,25S)-26-(β-D-Glucopyranosyloxy)-22-hydroxyfurostan-3-yl 6-O-(6-	949.5088, 903.4941, 757.4327, 595.3800	J, T	Steroidal saponins	/	[22]

							deoxy-α-L-mannopyranosyl)-β-D-glucopyranoside					
65	24.28	$[M+FA-H]^-$	949.5087	949.5014	7.7	$C_{45}H_{76}O_{18}$	Aspacochioside A	949.5087, 903.5010, 757.4414, 595.3938	J, T	Steroidal saponins	/	[25]
66	26.36	$[M-H]^-$	327.2191	327.2177	4.3	$C_{18}H_{32}O_5$	9,12,13-Trihydroxy-10,15-octadecadienoic acid	327.2191, 291.1928, 229.1477, 171.1036	J, H	Fatty acids	/	
67	26.80	$[M-H]^-$	1033.5331	1033.5225	10.3	$C_{50}H_{82}O_{22}$	Degalactotigonin	1033.5331, 901.4910, 871.4674, 739.4340, 577.3789	J, T	Steroidal saponins	/	[26]
68	27.05	$[M-H]^-$	1033.5325	1033.5225	9.7	$C_{50}H_{82}O_{22}$	Asparagoside F	1033.5325, 901.4907, 871.4788, 739.4320	J, T	Steroidal saponin	/	[27]
69	27.27	$[M+FA-H]^-$	1093.5540	1093.5436	9.5	$C_{51}H_{84}O_{22}$	Tribulosaponin B	1093.5540, 1047.5501, 901.4904, 885.4932, 739.4364	J	Steroidal saponins	/	[28]
70	27.56	$[M+FA-H]^-$	947.4927	947.4857	7.4	$C_{45}H_{74}O_{18}$	Terrestrosin A	947.4927, 901.4887, 739.4314, 577.3711	J	Steroidal saponins	/	[29]
71	27.85	$[M+FA-H]^-$	947.4928	947.4857	7.5	$C_{45}H_{74}O_{18}$	(3β,5α,25R)-Spirostan-3-yl O-β-D-galactopyranosyl-(1→2)-O-β-D-glucopyranosyl-(1→4)-β-D-galactopyranoside	947.4928, 901.4827, 739.4283, 577.3585	J, T	Steroidal saponins	/	[30]
72	28.46	$[M-H]^-$	329.2349	329.2333	4.9	$C_{18}H_{34}O_5$	9,12,13-Trihydroxy-10-octadecanoic acid	329.2349, 229.1463, 211.1354, 171.1042, 139.1135	J, H	Fatty acids	/	
73	28.57	$[M-H]^-$	1017.5361	1017.5276	8.4	$C_{50}H_{82}O_{21}$	(3β,5β,25S)-Spirostan-3-yl O-α-L-arabinopyranosyl-(1→4)-O-[6-deoxy-α-L-mannopyranosyl-(1→6)]-O-[β-D-glucopyranosyl-(1→2)]-β-D-glucopyranoside,	1017.5361, 885.4809, 739.4286, 577.3705	T	Steroidal saponins	/	[31]
74	28.73	$[M+FA-H]^-$	1075.5443	1075.5331	10.4	$C_{51}H_{82}O_{21}$	Pseudoprotodioscin	1075.5443, 1029.5375, 883.4792, 737.4056, 575.3477	J, H, T	Steroidal saponins	√	
75	29.02	$[M+FA-H]^-$	1075.5429	1075.5331	9.1	$C_{51}H_{82}O_{21}$	Pseudoprotoneodioscin	1075.5429, 1029.5391, 883.4711, 737.4204	J, H, T	Steroidal saponins	/	[32]
76	34.49	$[M+H]^+$	345.0980	345.0969	3.2	$C_{18}H_{16}O_7$	Boeravinone C	345.0980, 299.0916, 223.0594, 205.0498	X	Rotenoids	/	[33]

77	35.80	$[M-H]^-$	313.2397	313.2384	-0.4	$C_{18}H_{34}O_4$	12,13-Dihydroxy-9-octadecenoic acid	313.2397, 295.2266, 277.2187, 183.1416	H, T	Fatty acids	/	
78	35.87	$[M-H]^-$	313.2399	313.2379	1.6	$C_{18}H_{34}O_4$	9,10-Dihydroxy-9-octadecenoic acid	313.2399, 295.2269, 277.2161, 201.1122, 183.1384	H, T	Fatty acids	/	
79	36.18	$[M+H]^+$	520.3417	520.3403	-1.2	$C_{26}H_{50}NO_7P$	2-Linoleoyl-sn-glycero-3-phosphorylcholine	502.3417, 502.3331, 184.0742, 104.1064	H	Phospholipids	/	
80	36.29	$[M+H]^+$	520.3419	520.3421	-3.0	$C_{26}H_{50}NO_7P$	1-Linoleoyl-sn-glycero-3-phosphorylcholine	520.3419, 502.3308, 184.0741, 104.1068	H	Phospholipids	/	
81	36.45	$[M+H]^+$	496.3415	496.3421	-3.3	$C_{24}H_{50}NO_7P$	1-Palmitoyl-sn-glycero-3-phosphocholine	496.3415, 478.3186, 184.0727	T	Phospholipids	/	
82	36.60	$[M+H]^+$	496.3405	496.3421	-3.3	$C_{24}H_{50}NO_7P$	1-Palmitoylphosphatidylcholine	496.3399, 478.3324, 313.2725, 184.0734	J	Phospholipids	/	[34]
83	36.64	$[M-H]^-$	295.2290	295.2279	1.8	$C_{18}H_{32}O_3$	8-Hydroxy-11,14-octadecadienoic acid	295.2290, 277.2163, 195.1381, 171.1018	X	Fatty acids	/	
84	37.12	$[M-H]^-$	295.2291	295.2279	1.8	$C_{18}H_{32}O_3$	13-Hydroxy-9,11-octadecadienoic acid	295.2291, 277.2183, 195.1399	H, X	Fatty acids	/	

Note: "√" means these compounds were accurately identified by comparing with the standard substance database. "H" means "Huangjing", "X" means "Ximalayazimoli", "J" means "Jili", "T" means "Tiandong", "L" means "Xizanglengziqin".

Table S3. Top-ranked Degree value active ingredients.

Name	Degree	BetweennessCentrality	ClosenessCentrality
N-trans-Feruloyloctopamine	26	0.063113676	0.451428571
Terrestriamide	24	0.048167979	0.436464088
N-trans-Feruloyltyramine	23	0.044207918	0.431693989
12,13-Dihydroxy-9-octadecenoic acid	19	0.037467044	0.422459893
Anisodamine	14	0.018471765	0.393034826
9,12,13-Trihydroxy-10,15-octadecadienoic acid	13	0.014625657	0.385365854
N-trans-Caffeoyltyramine	11	0.010288131	0.385365854
Terrestribisamide	4	0.001235314	0.35426009
N-p-trans-Coumaroyltyramine	3	2.25E-04	0.351111111
N-trans-Feruloylmethoxytyramine	3	2.25E-04	0.351111111
Azelaic acid	2	2.23E-04	0.336170213
13-Hydroxy-9,11-octadecadienoic acid	1	0	0.32780083

Table S4. Network pharmacology analysis identified 67 MCODE genes in WGS.

NO.	Gene	NO.	Gene	NO.	Gene	NO.	Gene
1	AKT1	21	MDM2	41	ITGB1	61	CHEK1
2	TNF	22	FYN	42	NR3C1	62	FGFR1
3	EGFR	23	PRKACA	43	PPARA	63	AGTR1
4	ALB	24	JAK2	44	PRKCA	64	NOS3
5	CASP3	25	MMP2	45	LCK	65	ABCB1
6	BCL2	26	KIT	46	SYK	66	CSF1R
7	HIF1A	27	ABL1	47	FLT1	67	HDAC6
8	ESR1	28	PTPRC	48	AR		
9	MAPK3	29	PIK3R1	49	CDK1		
10	PPARG	30	BCL2L1	50	AKT2		
11	HSP90AA1	31	BRAF	51	SERPINE1		
12	MMP9	32	LYN	52	RPS6KB1		
13	PTGS2	33	PTPN11	53	RAF1		
14	HSP90AB1	34	PARP1	54	PIK3CB		
15	MAPK1	35	MCL1	55	BTK		
16	MTOR	36	MAPK14	56	ESR2		
17	KDR	37	CDK4	57	PIK3CG		
18	PIK3CA	38	HDAC1	58	TERT		
19	EP300	39	CDK2	59	PIK3CD		
20	GRB2	40	APP	60	BRD4		

Reference

1. Yao, X.; Deng, Z.; Li, H.; Zhang, B. Effect of processing cycles on the composition of Polygonatum cyrtonema Hua during nine-steam-nine-bask processing. Food Biosci. 2022, 50, https://doi.org/10.1016/j.fbio.2022.102081.
2. Wang, Q.; Ban, J.; Cai, R.; Zhang, X.; Lai, C.; Chen, Y.; Li, X.; Chen, C.; Chen, Y.; Zhang, Z.; et al. Metabolic Composition and Quality Traits of Polygonatum cyrtonema Hua from Different Germplasms and Age Sections Based on Widely Targeted Metabolomics Analysis. Int. J. Mol. Sci. 2023, 24, 6077, https://doi.org/10.3390/ijms24076077.
3. Yang, L.; Kang, Y.; Li, N.; Wang, Y.; Mou, H.; Sun, H.; Ao, T.; Chen, L.; Chen, W. Unlocking hormesis and toxic effects induced by cadmium in Polygonatum cyrtonema Hua based on morphology, physiology and metabolomics. J. Hazard. Mater. 2024, 465, 133447, https://doi.org/10.1016/j.jhazmat.2024.133447.
4. Kisby, G.E.; Moore, H.; Spencer, P.S. Animal models of brain maldevelopment induced by cycad plant genotoxins. Birth Defects Res. Part C: Embryo Today: Rev. 2013, 99, 247–255, https://doi.org/10.1002/bdrc.21052.
5. Zhou, Y.; Jiang, S.; Li, C.; Peng, S. Chemical constituents of Cycas panzhihuaensis. Chinese Journal of Applied and Environment Biology 1999, 4, 34–37.
6. Chiavaroli, A.; Di Simone, S.C.; Acquaviva, A.; Nilofar, N.; Libero, M.L.; Brunetti, L.; Recinella, L.; Leone, S.; Orlando, G.; Zengin, G.; et al. Neuromodulatory Effects Induced by the Association of Moringa oleifera Lam., Tribulus terrestris L., Rhodiola rosea Lam., and Undaria pinnatidifida Extracts in the Hypothalamus. Chem. Biodivers. 2024, 21, e202302075, https://doi.org/10.1002/cbdv.202302075.
7. Yang, X.; Guan, Y.; Yan, B.; Xie, Y.; Zhou, M.; Wu, Y.; Yao, L.; Qiu, X.; Yan, F.; Chen, Y.; et al. Evidence-based complementary and alternative medicine bioinformatics approach through network pharmacology and molecular docking to determine the molecular mechanisms of Erjing pill in Alzheimer's disease. Exp. Ther. Med. 2021, 22, 1–13, https://doi.org/10.3892/etm.2021.10687.
8. Wang, S.; Du, D.; Li, F.; Chen, M.; Sheng, H.; Zhang, C.; Guo, F.; Chen, Z.; Cao, G. “UHPLC‐Q‐TOF/MS‐chemometrics‐network pharmacology” integrated strategy to discover quality markers of raw and stir‐fried Fructus Tribuli and process optimization of stir‐fried Fructus Tribuli. Phytochem. Anal. 2024, 35, 1036–1051, https://doi.org/10.1002/pca.3339.
9. Bhutani, S.P.; Chibber, S.S.; Seshadri, T.R. Flavonoids of the fruits and leaves of Tribulus terrestris: Constitution of tribuloside. Phytochemistry 1969, 8, 299–303.
10. Zhang, G.-L.; Xing, Q.-Y.; Zhang, M.-Z. Glycolipids from Mirabilis himalaica. Phytochemistry 1997, 45, 1213–1215, https://doi.org/10.1016/S0031-9422(97)00114-3.
11. Qi, Q.; Yu, Y.; Tang, C. Screening of the potentially active compounds from Polygonatum sibiricum using RAW264.7 cellular membranes coated magnetic beads fishing followed by HPLC analysis. Biomed. Chromatogr. 2019, 34, e4763, https://doi.org/10.1002/bmc.4763.
12. Xue, X.; Jiao, Q.; Jin, R.; Wang, X.; Li, P.; Shi, S.; Huang, Z.; Dai, Y.; Chen, S. The combination of UHPLC-HRMS and molecular networking improving discovery efficiency of chemical components in Chinese Classical Formula. Chin. Med. 2021, 16, 50, https://doi.org/10.1186/s13020-021-00459-6.

13. Liao, W.C.; Huang, J.-P.; Huang, W.-Y. Chemical composition analysis and biofunctionality of Polygonatum sibiricum and Polygonatum odoratum extracts. BioResources 2023, 18, 3608–3619, https://doi.org/10.15376/biores.18.2.3608-3619.
14. Li, J.; Wang, Z.; Fan, M.; Hu, G.; Guo, M. Potential Antioxidative and Anti-Hyperuricemic Components Targeting Superoxide Dismutase and Xanthine Oxidase Explored from Polygonatum Sibiricum Red.. Antioxidants 2022, 11, 1651, https://doi.org/10.3390/antiox11091651.
15. Jian, R.; Zeng, K.-W.; Li, J.; Li, N.; Jiang, Y.; Tu, P. Anti-neuroinflammatory constituents from Asparagus cochinchinensis. Fitoterapia 2013, 84, 80–84, https://doi.org/10.1016/j.fitote.2012.10.011.
16. Pang, X.; Gao, L.; Wang, B.; Chen, X.-J.; Zhang, J.; Guo, B.-L.; Ma, B.-P. New steroidal glycosides from the roots of Asparagus cochinchinensis. J. Asian Nat. Prod. Res. 2021, 23, 205–216, https://doi.org/10.1080/10286020.2021.1873956.
17. Wu, T.-S.; Shi, L.-S.; Kuo, S.-C. Alkaloids and other constituents from Tribulus terrestris. Phytochemistry 1999, 50, 1411–1415, https://doi.org/10.1016/s0031-9422(97)01086-8.
18. Shen, Y.; Chen, H.S.; Wang, Q. Studies on chemical constituents of Asparagus cochinchinensis. Academic Journal of Second Military Medical University 2007, 28, 1241–1244.
19. Evstatieva, L.; Tchorbanov, B. Complex Investigations ofTribulus TerrestrisL. for Sustainable use by Pharmaceutical Industry. Biotechnol. Biotechnol. Equip. 2011, 25, 2341–2347, https://doi.org/10.5504/bbeq.2011.0035.
20. Meng, X.; Xing, J.; Liu, S.; Liu, Z.; Song, F. Comprehensive chemical profiling and potential chemical marker's evaluation of Tribulus terrestris by UPLC-QTOF-MS in combination with ion mobility spectrometry. J. Pharm. Biomed. Anal. 2022, 217, 114839, https://doi.org/10.1016/j.jpba.2022.114839.
21. Nagahama, T. Studies on Neocycasins, New Glycosides of Cycads. Bulletin of the Faculty of Agriculture, Kagoshima University 1964, 14, 1–50.
22. Konishi, T.; Shoji, J. Studies on the constituents of asparagi radix. I. On the structures of furostanol oligosides of Asparagus cochinchinensis (Loureio) Merrill.. *Chem. Pharm. Bull.* **1979**, *27*, 3086–3094, https://doi.org/10.1248/cpb.27.3086.
23. Sautour, M.; Miyamoto, T.; Lacaille-Dubois, M.-A. Steroidal saponins from Asparagus acutifolius. Phytochemistry 2007, 68, 2554–2562, https://doi.org/10.1016/j.phytochem.2007.02.033.
24. Agrawal, P.K. Dependence of 1H NMR chemical shifts of geminal protons of glycosyloxy methylene (H2‐26) on the orientation of the 27‐methyl group of furostane‐type steroidal saponins. Magn. Reson. Chem. 2004, 42, 990‐993, https://doi.org/10.1002/mrc.1474.
25. Yang, Y.C.; Huang, S.Y.; Shi, J.G. Two new furostanol glycosides from Asparagus cochinchinensis. Chinese Chemical Letters 2002, 13, 1185–1188,
26. Kang, L.P.; Wu, K.L.; Yu, H.S.; Pang, X.; Liu, J.; Han, L.F.; Zhang, J.; Zhao, Y.; Xiong, C.Q.; Song, X.B.; et al. Steroidal saponins from Tribulus terrestris. Phytochemistry 2014, 107, 182–189.
27. Xue, X.; Jin, R.; Jiao, Q.; Li, X.; Li, P.; Shen, G.; Shi, S.; Huang, Z.; Dai, Y.; Zhang, S. Differentiation of three Asparagus species by UHPLC-MS/MS based molecular networking identification and chemical profile analysis. J. Pharm. Biomed. Anal. 2022, 219, 114863, https://doi.org/10.1016/j.jpba.2022.114863.

28. Bedir, E.; Khan, I.A. New Steroidal Glycosides from the Fruits of Tribulus terrestris. J. Nat. Prod. 2000, 63, 1699–1701, https://doi.org/10.1021/np000353b.
29. Uysal, S.; Senkardes, I.; Jekő, J.; Cziáky, Z.; Zengin, G. Chemical characterization and pharmacological profile of Tribulus terrestris extracts: A novel source of cosmeceuticals and pharmaceuticals. Biochem. Syst. Ecol. 2023, 107, https://doi.org/10.1016/j.bse.2023.104600.
30. Mohamed, M.A. Spirostanol Saponins from Asparagus sprengeri and Their Molluscicidal Activity. Natural Product Communications 2007, 2, 731–736, https://doi.org/10.1177/1934578X0700200705.
31. Jaiswal, Y.; Liang, Z.; Ho, A.; Chen, H.; Zhao, Z. A Comparative Tissue‐specific Metabolite Analysis and Determination of Protodioscin Content in Asparagus Species used in Traditional Chinese Medicine and Ayurveda by use of Laser Microdissection, UHPLC‐QTOF/MS and LC–MS/MS. Phytochem. Anal. 2014, 25, 514–528, https://doi.org/10.1002/pca.2522.
32. Ling, Y.; He, X.; Jiang, R.; Zhang, Q.; Yuan, S.; Liang, Y.; Li, C.; Zhao, Y.; Zhang, Q.; Liu, K. Rapid Detection and Characterization of Steroidal Saponins in the Root of Asparagus cochinchinensis by High-Performance Liquid Chromatography Coupled to Electrospray Ionization and Quadrupole Time-of-Flight Mass Spectrometry. J. Chromatogr. Sci. 2020, 58, 454–463, https://doi.org/10.1093/chromsci/bmaa006.
33. Eizirik, D.; Kisby, G. Cycad toxin-induced damage of rodent and human pancreatic β-cells. Biochem. Pharmacol. 1995, 50, 355–365, https://doi.org/10.1016/0006-2952(95)00150-x.
34. Wang, Y.; Guo, W.; Xie, S.; Liu, Y.; Xu, D.; Chen, G.; Xu, Y. Multi-omics analysis of brain tissue metabolome and proteome reveals the protective effect of gross saponins of Tribulus terrestris L. fruit against ischemic stroke in rat. J. Ethnopharmacol. 2021, 278, 114280, https://doi.org/10.1016/j.jep.2021.114280.
